# Supplementary figures and images for: Cell shape anisotropy contributes to self-organized feather pattern fidelity in birds
Source: PLoS Biol. 2022 Oct 10;20(10):e3001807. doi: 10.1371/journal.pbio.3001807 (PMC9584522; doi:10.1371/journal.pbio.3001807)

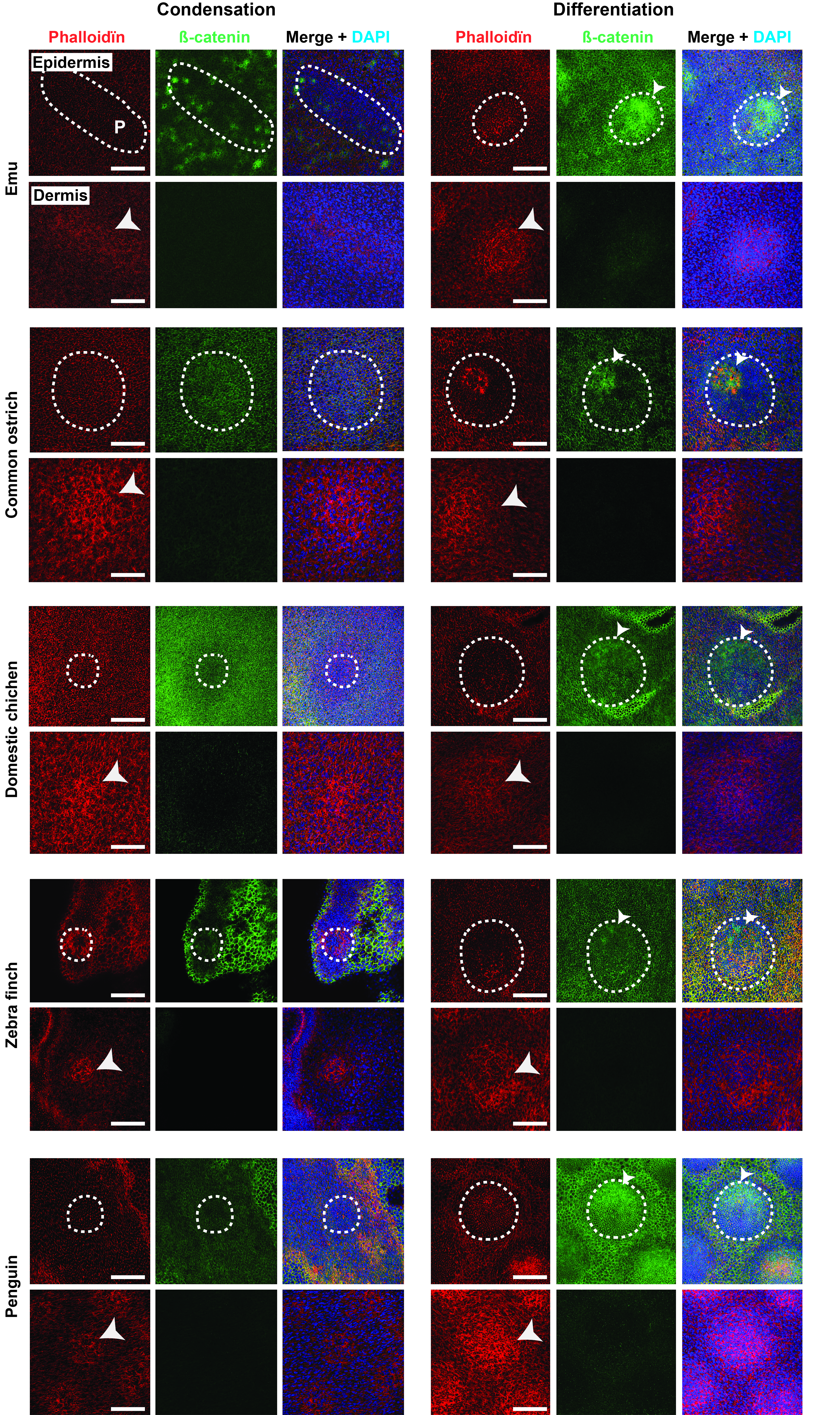

Supplement: S1 Fig — The 40× confocal views located within competent skin regions of emu, common ostrich, domestic chicken, zebra finch, and penguin flat skins and oriented along the antero-posterior axis, show DAPI (in blue), β–catenin (in green), and phalloïdin (in red) stains. Similarly to the Japanese quail (see Fig 1), epidermal and dermal cells compacted locally in primordia (P, white dotted lines in the epidermis, arrowhead in the dermis) at condensation stage and initiated programs of feather production upon nuclear translocation of β-catenin in epidermal nuclei (white arrows) at differentiation stage. Scale bars: 100 μm. (JPG) [file pbio.3001807.s001.jpg]

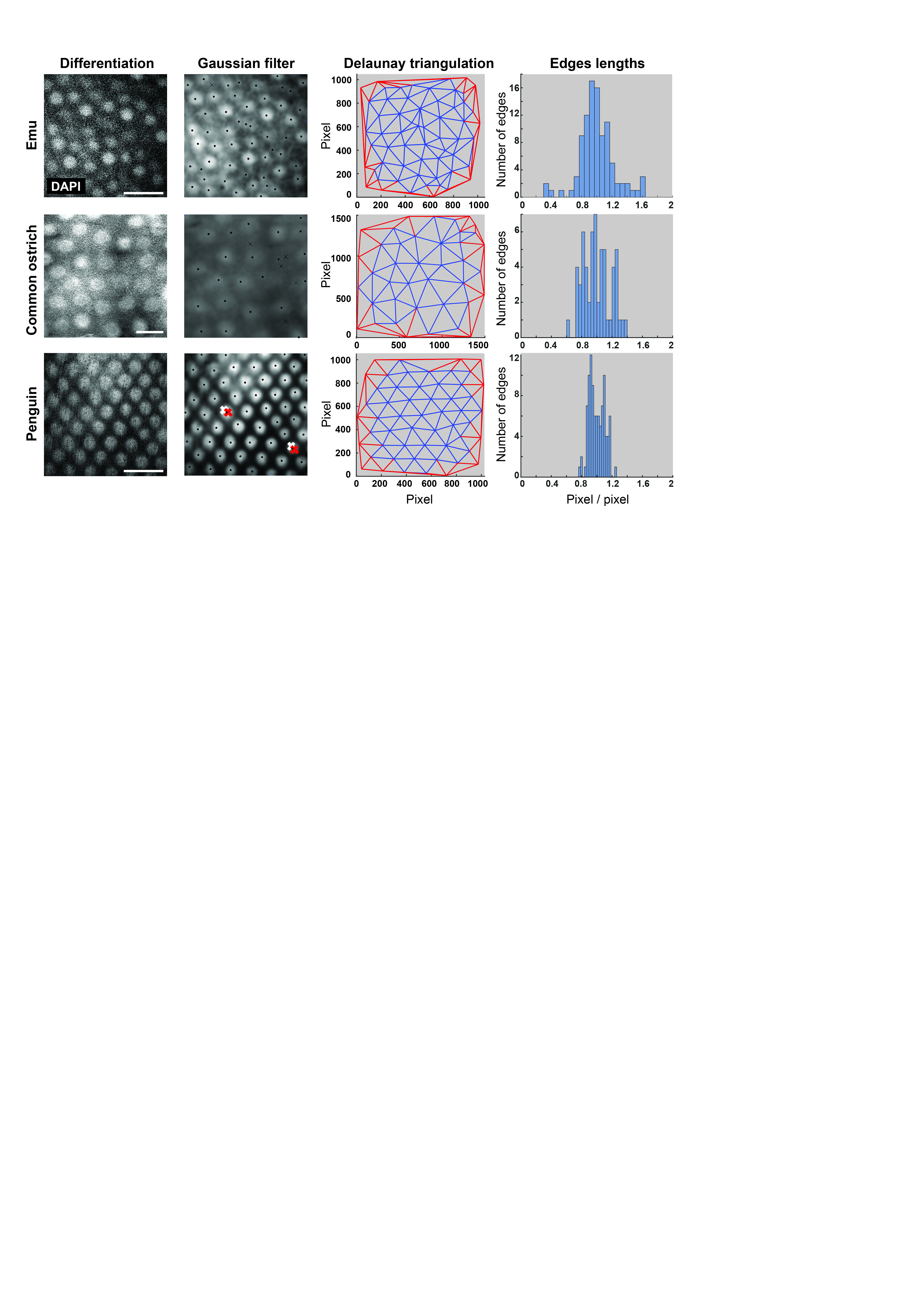

Supplement: S2 Fig — The 10× confocal views of DAPI stains (in white) in flat embryonic skins of emu, common ostrich, and penguin at differentiation stage and associated positions of feather primordia centers (black dots) detected by applying a custom-made MATLAB program (Dotfinder; [26]) manually corrected in a few cases (crosses) allowed obtaining Delaunay triangulation representations. Triangles edges shown in red possess 1 vertex on the image boundary and were ignored in the analysis. Histograms show the distributions of edge lengths for each species (y-axis: number of edges) and illustrate spacing variability, quantified as standard deviation of normalized edge lengths (see Materials and methods and Fig 2). The data underlying this figure can be found at 10.5281/zenodo.7006365. Scale bar: 500 μm. (JPG) [file pbio.3001807.s002.jpg]

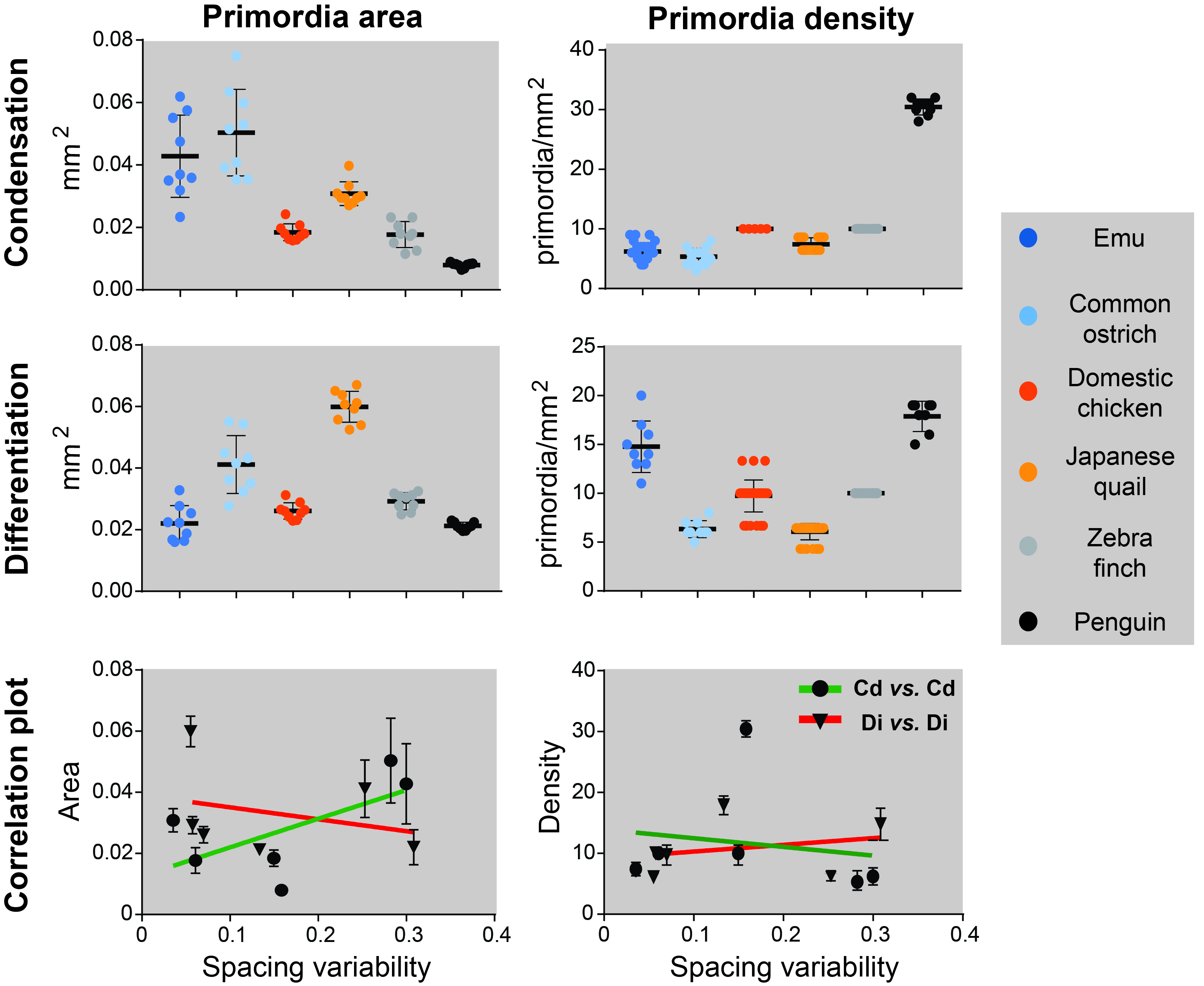

Supplement: S3 Fig — Quantifications of feather primordia size (in mm2) and density (in primordia/mm2) at condensation and differentiation stages are shown for each color-coded each species. Plotting spacing variability values vs. average primordia area or density at condensation stage (Cd; dots and green line; Pearson’s correlation coefficient r = 0.6295 and 0.1654) and differentiation stage (Di; triangles and red line; r = 0.2865 and 0.2575) showed that spacing variability is not correlated to primordia size or density. The data underlying this figure can be found at 10.5281/zenodo.7006365. Error bars: mean with standard deviation. (JPG) [file pbio.3001807.s003.jpg]

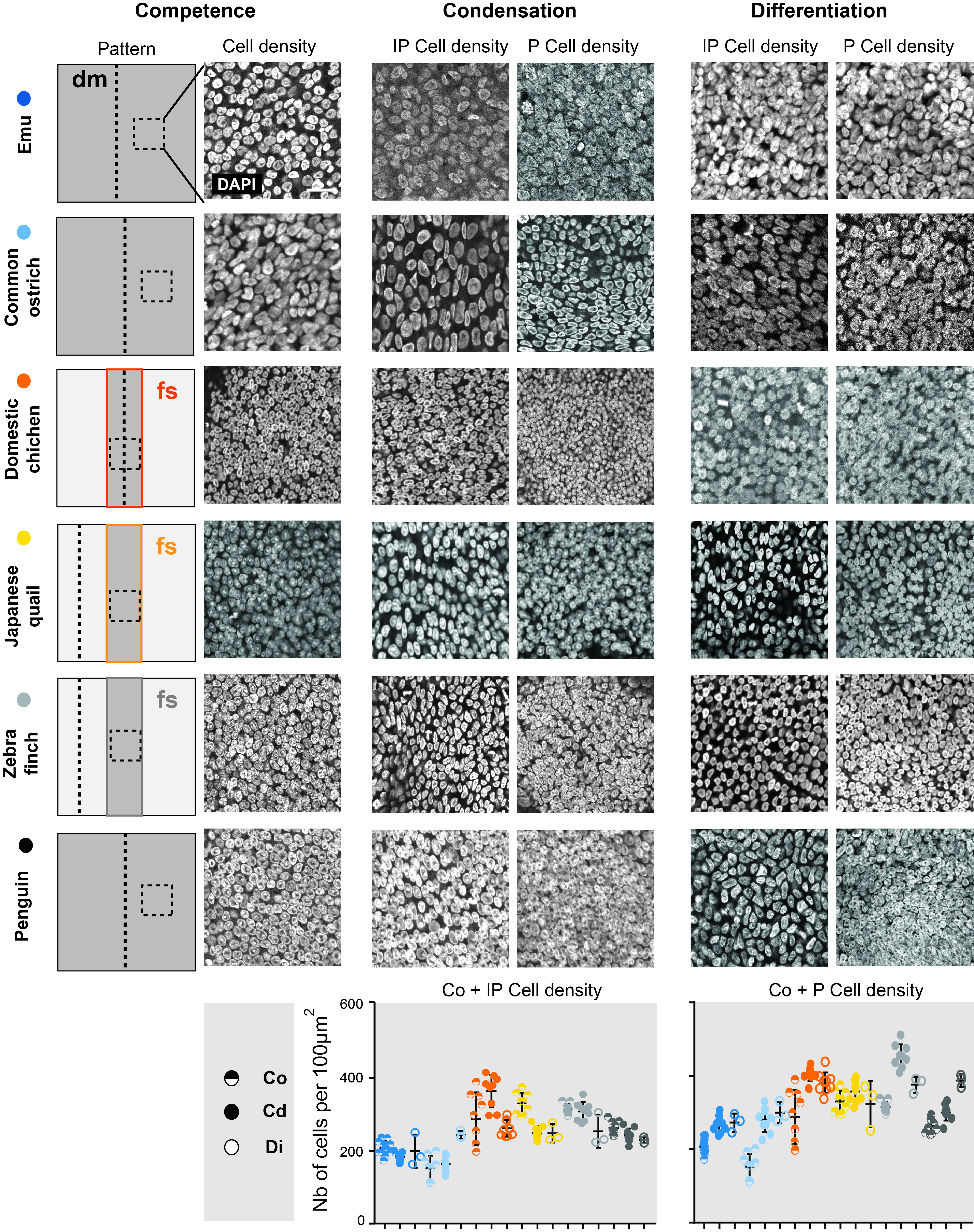

Supplement: S4 Fig — The 40× confocal views at epidermal levels of DAPI stains (in white) in flat embryonic skins of emu, common ostrich, domestic chicken, Japanese quail, and penguin are shown at competence stage (Co) together with corresponding schematics (black dotted squares show the position of images and see Fig 3), as well as at condensation (Cd) and differentiation (Di) stages in the inter-primordium (IP) and primordium (P) region. Quantifications of cell densities are shown in corresponding graphs for competent stage (Co, bicolored circles primordia, in both graphs) and for condensation (Cd, dots) and differentiation (Di, circles) in primordia (left graph) and inter-primordia (right graph) regions. Cell density increased through time, inter-species variation appearing largely independent of tissue level or stage. The data underlying this figure can be found at 10.5281/zenodo.7006365. Scale bar: 20 μm. Error bars: mean with standard deviation. (JPG) [file pbio.3001807.s004.jpg]

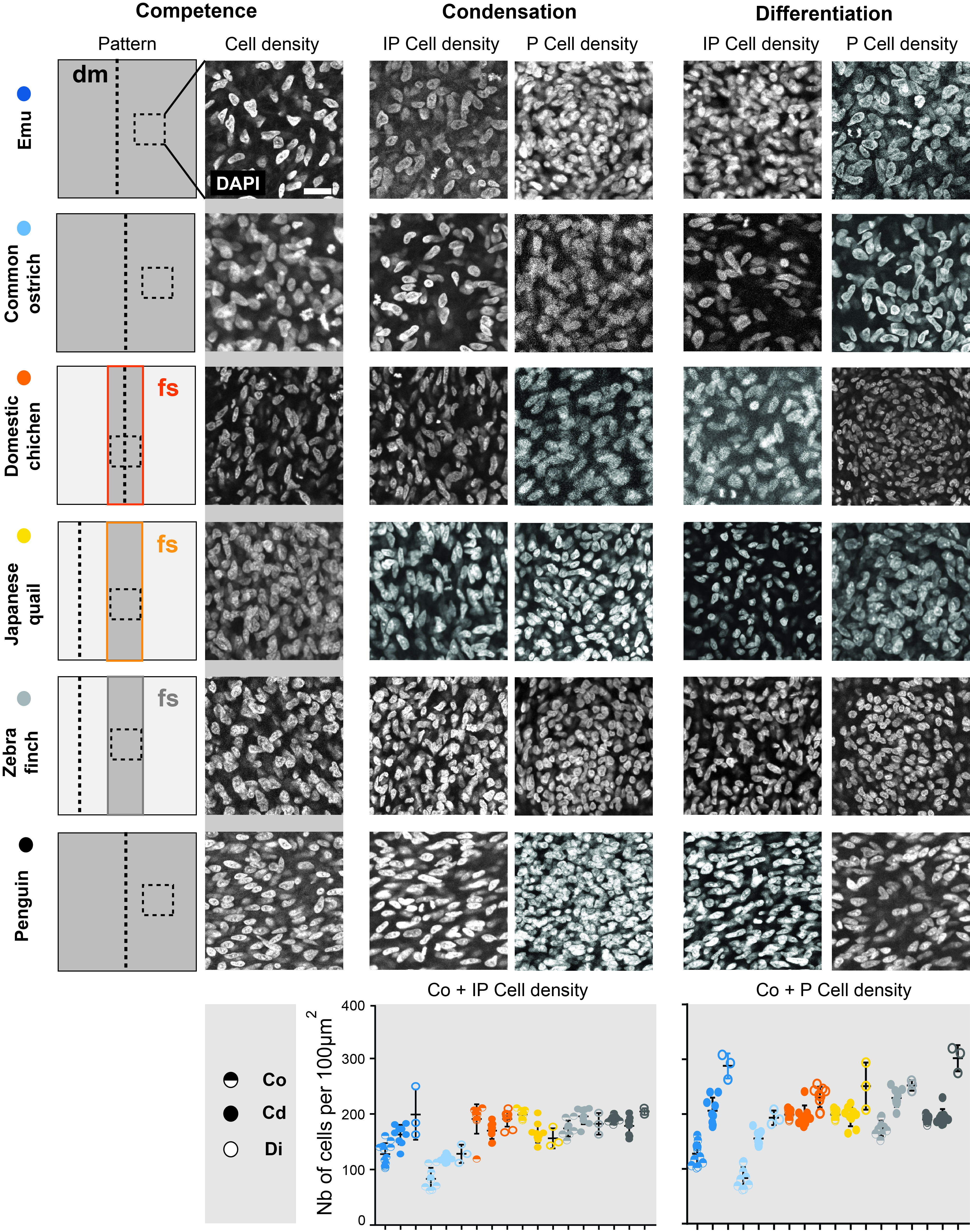

Supplement: S5 Fig — The 40× confocal views at dermal levels of DAPI stains (in white) in flat embryonic skins of emu, common ostrich, domestic chicken, Japanese quail, and penguin are shown at competence stage (Co) together with corresponding schematics (black dotted squares show the position of images and see Fig 3), as well as at condensation (Cd) and differentiation (Di) stages in the inter-primordium (IP) and primordium (P) region. Quantifications of cell densities are shown in corresponding graphs for competent stage (Co, bicolored circles primordia, in both graphs) and for condensation (Cd, dots) and differentiation (Di, circles) in primordia (left graph) and inter-primordia (right graph) regions. Cell density increased through time, inter-species variation appearing largely independent of tissue level or stage. The data underlying this figure can be found at 10.5281/zenodo.7006365. Scale bar: 20 μm. Error bars: mean with standard deviation. (JPG) [file pbio.3001807.s005.jpg]

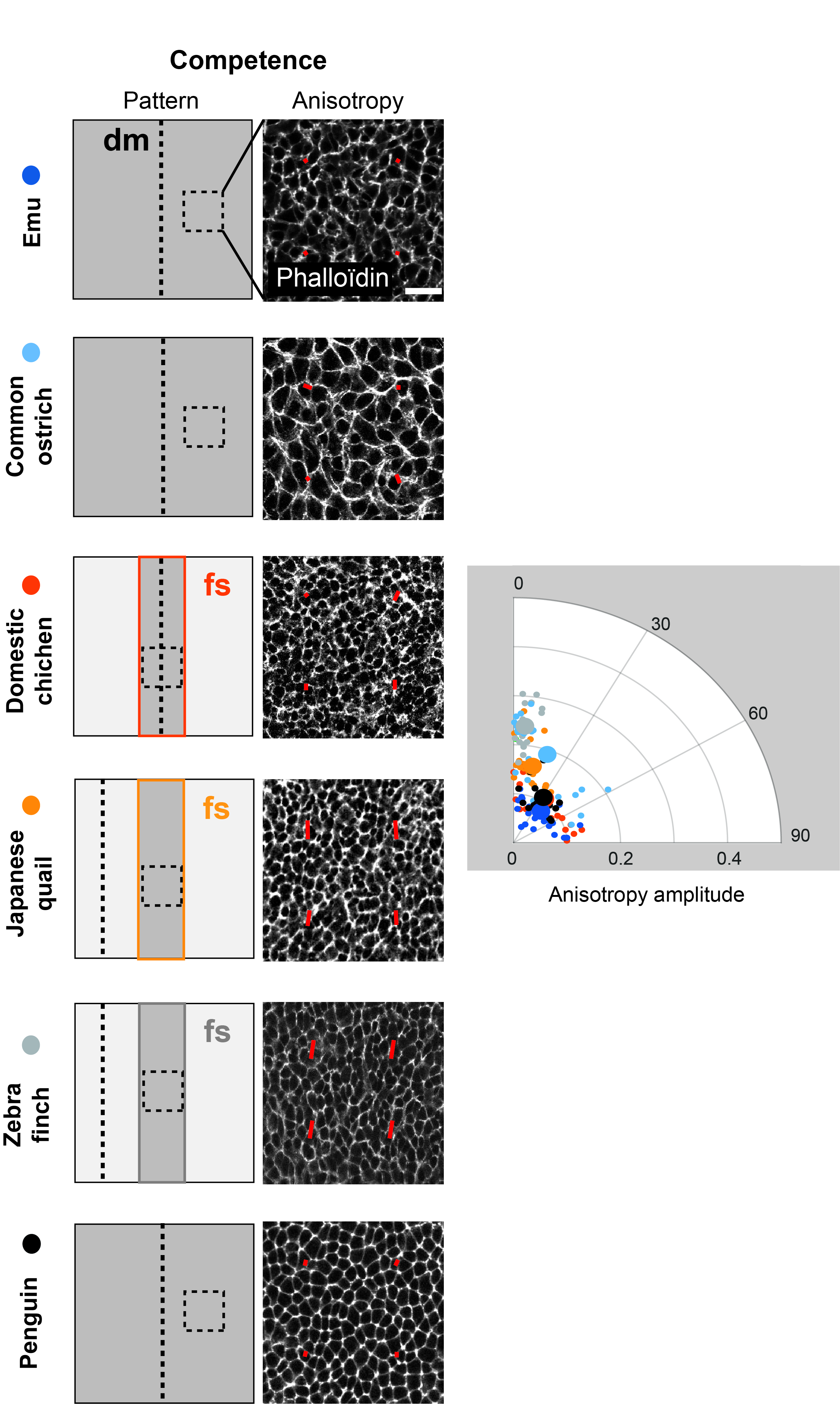

Supplement: S6 Fig — Left panels: 40× confocal views of 100 μm2 magnifications of phalloïdin stains (in white) in inter-primordia regions of the epidermis on flat embryonic skins of each species at competence stage and corresponding schematics indicating the position of images (black dotted squares) show the anisotropy of average cell shapes (as described in Fig 3; red bars). Scale bar: 20 μm. Right panel: Quantifications of anisotropy amplitude in color-coded species are represented into polar coordinates for each stage (small dots are individual values, large dots are averaged values; n = 3 specimen per species). The data underlying this figure can be found at 10.5281/zenodo.7006365. (JPG) [file pbio.3001807.s006.jpg]

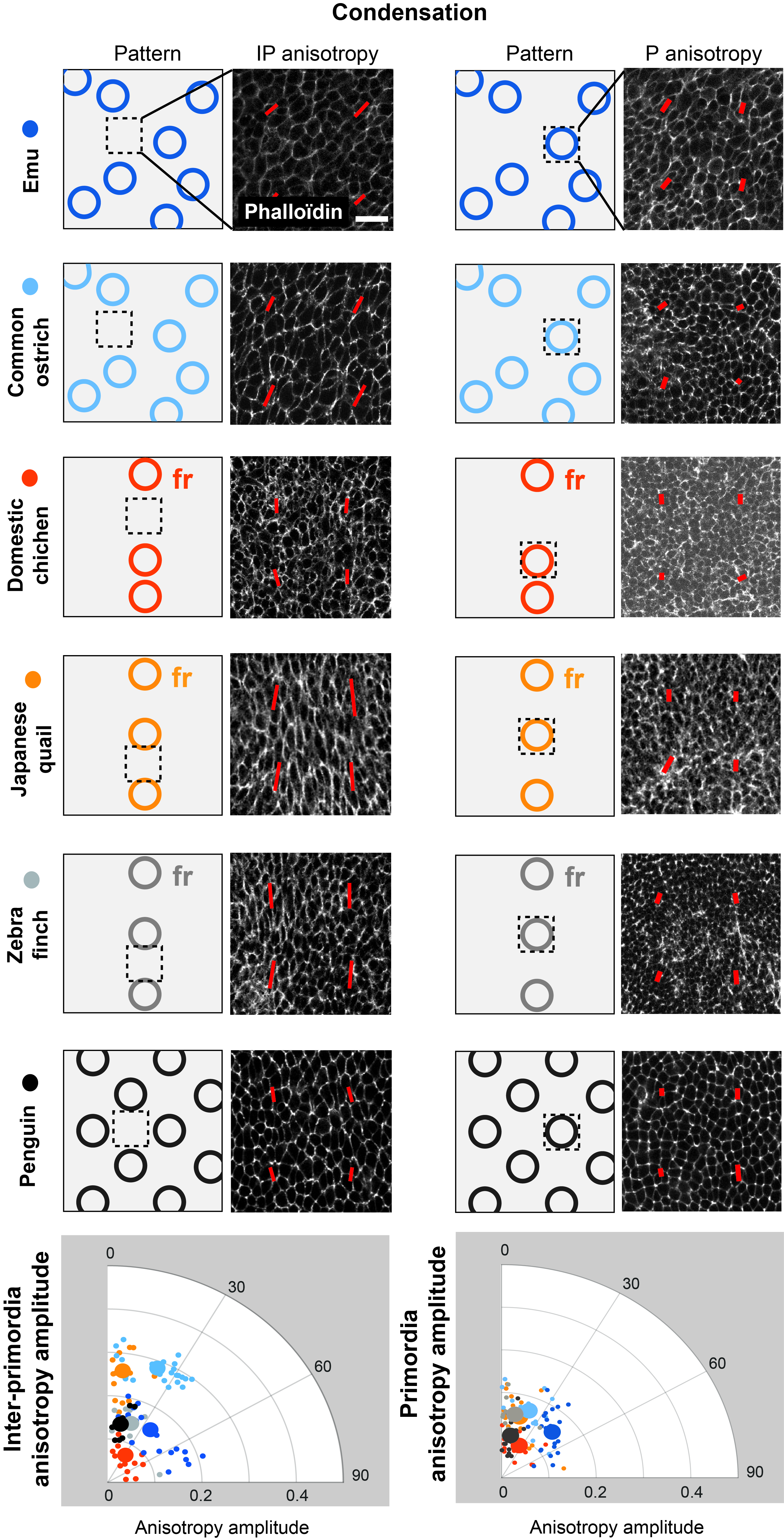

Supplement: S7 Fig — The 40× confocal views of 100 μm2 magnifications of phalloïdin stains (in white) in inter-primordia (left column) and primordia (right column) regions of the epidermis on flat embryonic skins of each species at condensation stage and corresponding schematics indicating the position of images (black dotted squares) show the anisotropy of average cell shapes (as described in Fig 3; red bars). Scale bar: 20 μm. Quantifications of anisotropy amplitude in color-coded species are represented into polar coordinates for each stage (small dots are individual values, large dots are averaged values; n = 3 specimen per species). The data underlying this figure can be found at 10.5281/zenodo.7006365. (JPG) [file pbio.3001807.s007.jpg]

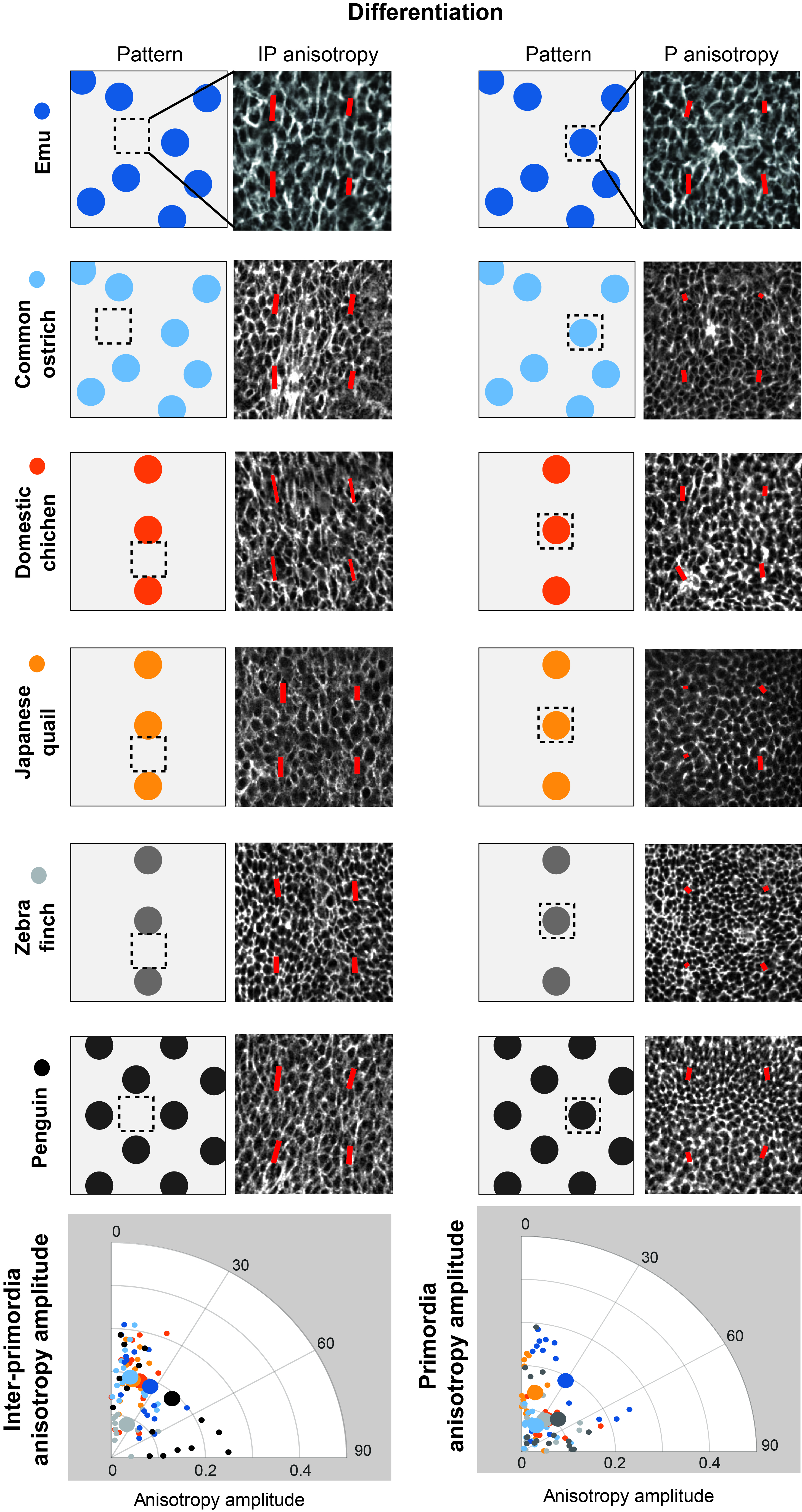

Supplement: S8 Fig — The 40× confocal views of 100 μm2 magnifications of phalloïdin stains (in white) in inter-primordia (left column) and primordia (right column) regions of the epidermis on flat embryonic skins of each species at differentiation stage and corresponding schematics indicating the position of images (black dotted squares) show the anisotropy of average cell shapes (as described in Fig 3; red bars). Scale bar: 20 μm. Quantifications of anisotropy amplitude in color-coded species are represented into polar coordinates for each stage (small dots are individual values, large dots are averaged values; n = 3 specimen per species). The data underlying this figure can be found at 10.5281/zenodo.7006365. (JPG) [file pbio.3001807.s008.jpg]

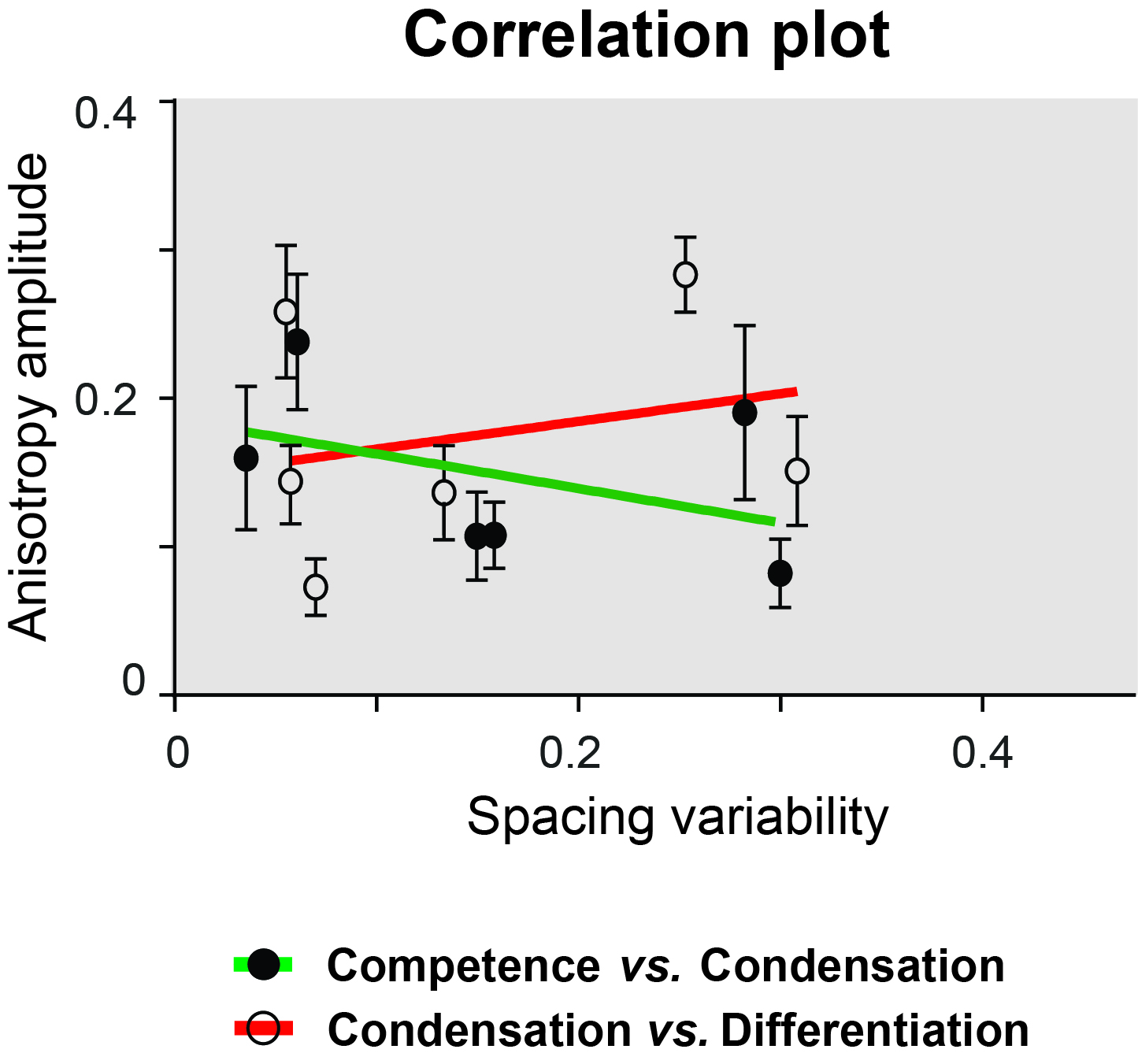

Supplement: S9 Fig — The plot shows non-correlating averaged values of epidermal cell anisotropy at competence stage vs. spacing variability at condensation stage (black dots and green line; Pearson’s correlation coefficient r = −0.4274) or at condensation stage vs. differentiation stage (circles and red line; r = 0.2543). The data underlying this figure can be found at 10.5281/zenodo.7006365. Error bars: standard deviation. (JPG) [file pbio.3001807.s009.jpg]

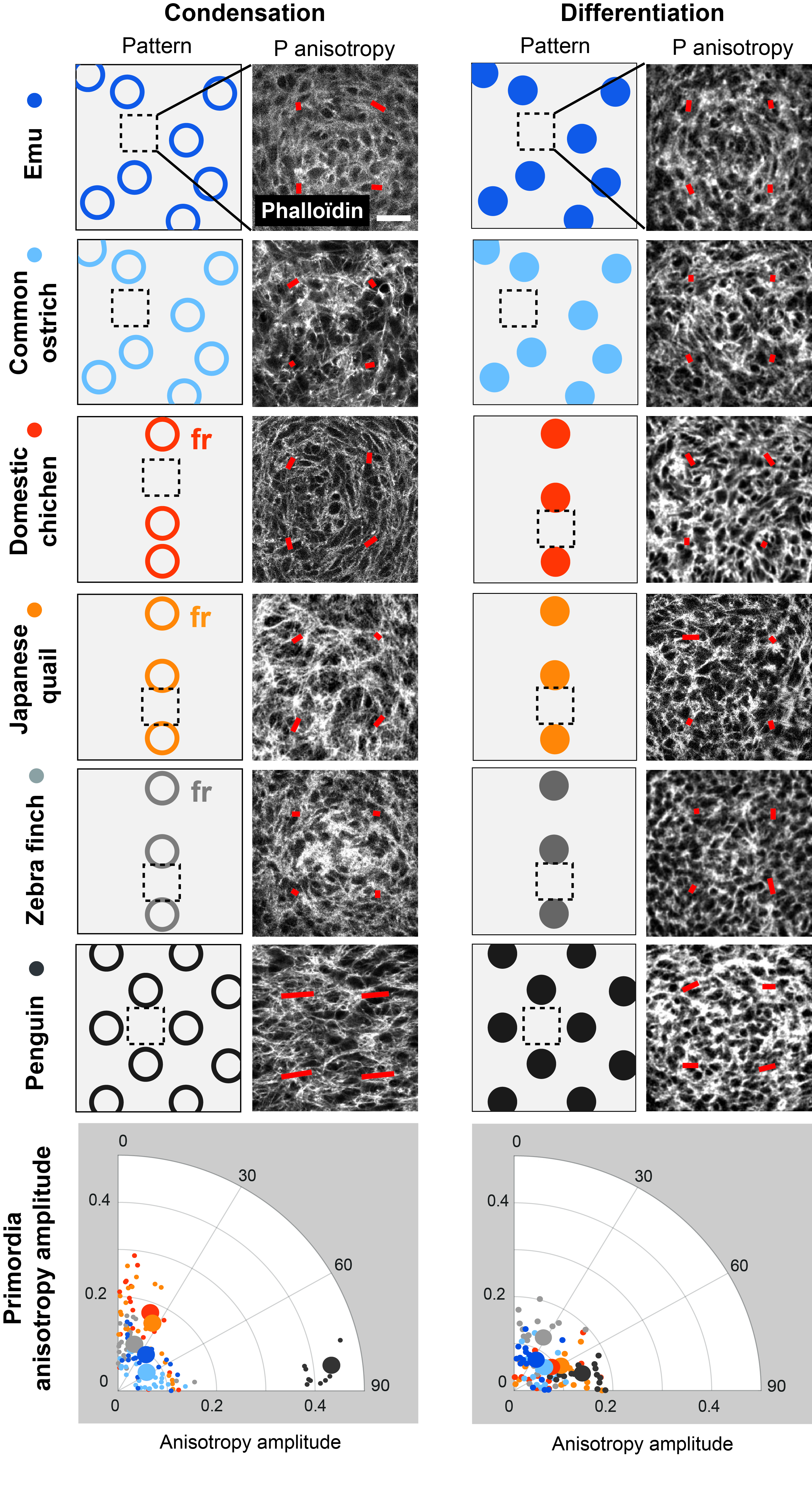

Supplement: S10 Fig — The 40× confocal views of 100 μm2 magnifications of phalloïdin stains (in white) primordia regions on flat embryonic skins of each species at condensation stage (left column) and differentiation stage (right column) and corresponding schematics indicating the position of images (black dotted squares) show the anisotropy of average cell shapes (as described in Fig 3; red bars). Scale bar: 20 μm. Quantifications of anisotropy amplitude in color-coded species are represented into polar coordinates for each stage (small dots are individual values, large dots are averaged values; n = 3 specimen per species). The data underlying this figure can be found at 10.5281/zenodo.7006365. (JPG) [file pbio.3001807.s010.jpg]

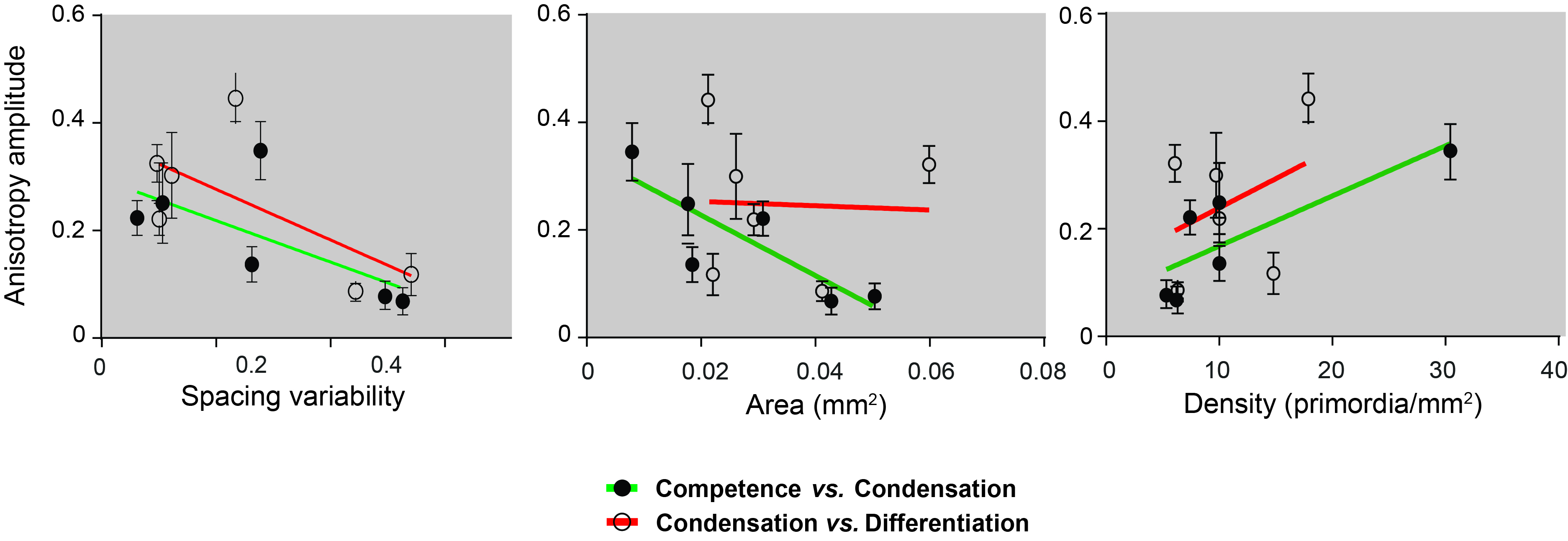

Supplement: S11 Fig — The left plot shows correlating averaged values of dermal cell anisotropy at competence stage vs. spacing variability at condensation stage (black dots and green line; Pearson’s correlation coefficient r = −0.6769) or at condensation stage vs. differentiation stage (circles and red line; r = −0.6689). Middle and right plots show non-correlating values of averaged dermal cell anisotropy amplitude at competence stage vs. primordia area or density at condensation stage (black dots and green line; Pearson’s correlation coefficients r = −0.84305 or −0.8039) or at condensation stage vs. differentiation stage (circles and red line; r = 0.04398 or 0.3800). The data underlying this figure can be found at 10.5281/zenodo.7006365. Error bars: standard deviation. (JPG) [file pbio.3001807.s011.jpg]

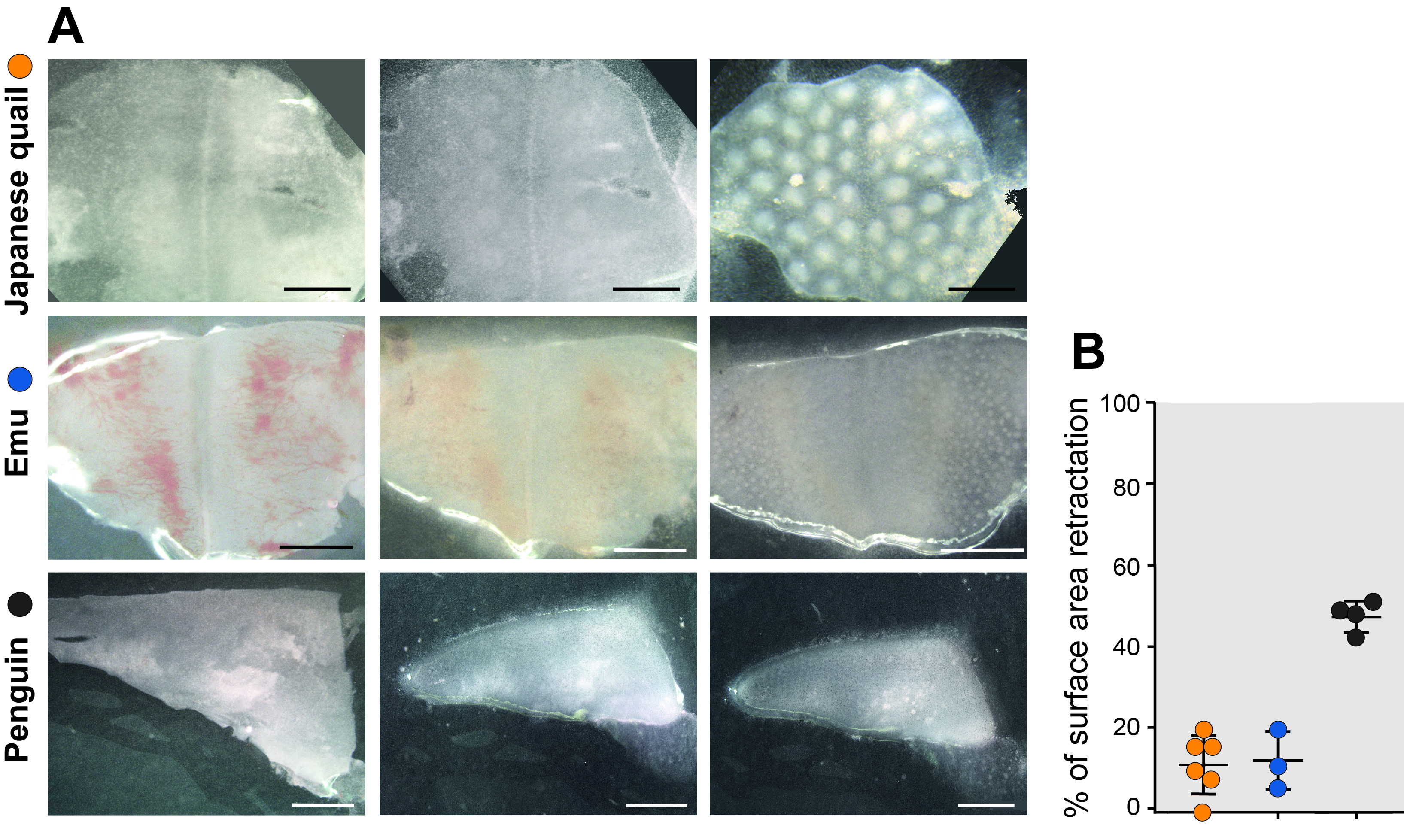

Supplement: S12 Fig — (A) Dorsal skin explants of Japanese quail, emu, and African penguin embryos prepared at competence stage recapitulate timely dynamics of primordia emergence. Scale bars: 2 mm. (B) Quantifications of the percentage of surface area retractation at competence and differentiation stages (see Materials and methods) show that Japanese quail, emu, and penguin explants shrink by approximately 10%, 10%, and 50% of their surface, respectively. The data underlying this figure can be found at 10.5281/zenodo.7006365. (JPG) [file pbio.3001807.s012.jpg]

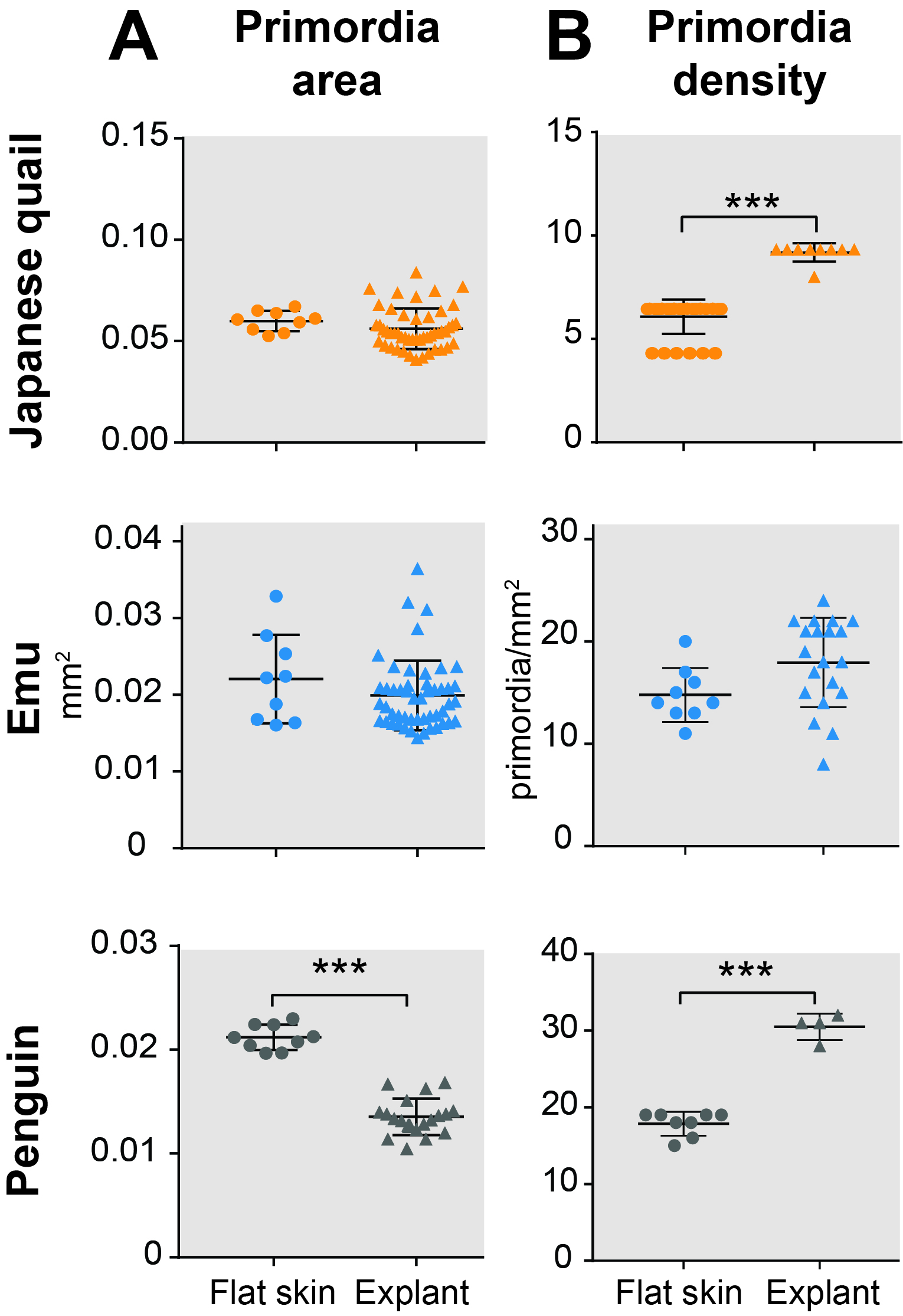

Supplement: S13 Fig — (A) Quantifications of primordia size (in mm2) at differentiation stage showed no significant difference between control flat skins and cultured explants in the Japanese quail (unpaired 2-tailed t test; p = 0.2776) and the emu (p = 0.2180) and but a significant reduction in the penguin (p < 0.0001). (B) Density (in primordia/mm2) was conserved in the emu (p = 0.0543) but significantly increased in the Japanese quail (p < 0.0001) and penguin (p < 0.0001). The data underlying this figure can be found at 10.5281/zenodo.7006365. Error bars: mean with standard deviation; significance of statistical tests is shown with stars. (JPG) [file pbio.3001807.s013.jpg]

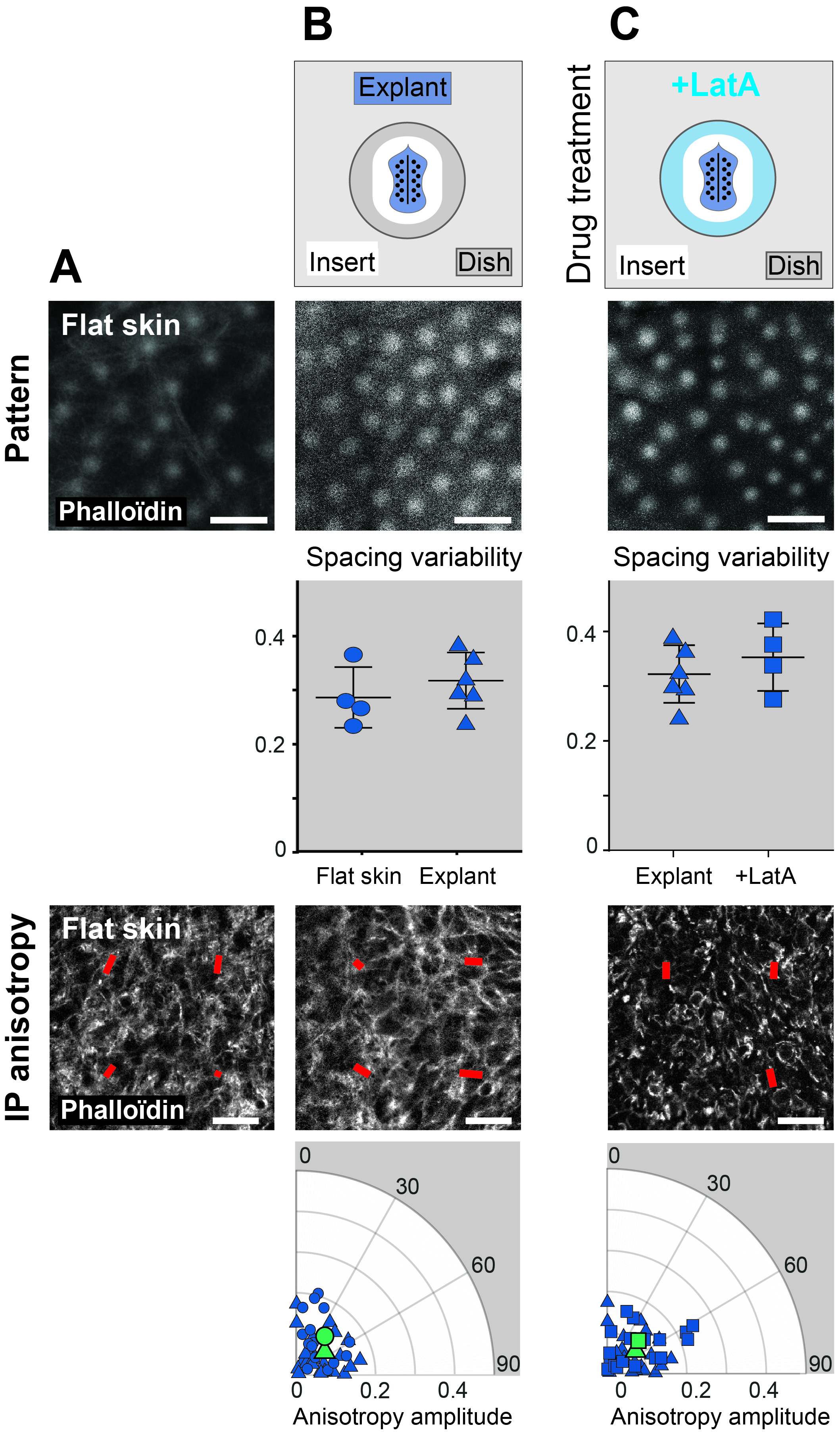

Supplement: S14 Fig — (A) The 3,2× (upper panel) and 40× (bottom panel) views of a phalloïdin-stained emu flat skin. (B) Dissected portions of dorsal emu embryonic skin (i.e., explant, in blue) were cultured to differentiation stage (see Fig 4). Quantifications of spacing variability show that at that stage, pattern fidelity was maintained in explants (triangles; n = 6) compared to flat skis (dots; n = 4; unpaired 2-tailed t test; p = 0.3963). (C) Emu skin explants were treated with low-doses of Latrunculin A (LatA, in blue) and cultured to differentiation stage (see Fig 5). Quantifications of spacing variability showed that at this stage, low pattern fidelity was maintained (squares; n = 5; p = 0.4195) compared to control cultured explants (triangles; n = 5). Confocal views and respective quantifications (as described in Fig 3) of phalloïdin-stained inter-primordia dermis showed that cells remain isotropic, which is typical of this species at this stage. The data underlying this figure can be found at 10.5281/zenodo.7006365. Error bars: mean with standard deviation. Scale bars: 500 μm (primordia pattern), 20 μm (anisotropy). (JPG) [file pbio.3001807.s014.jpg]

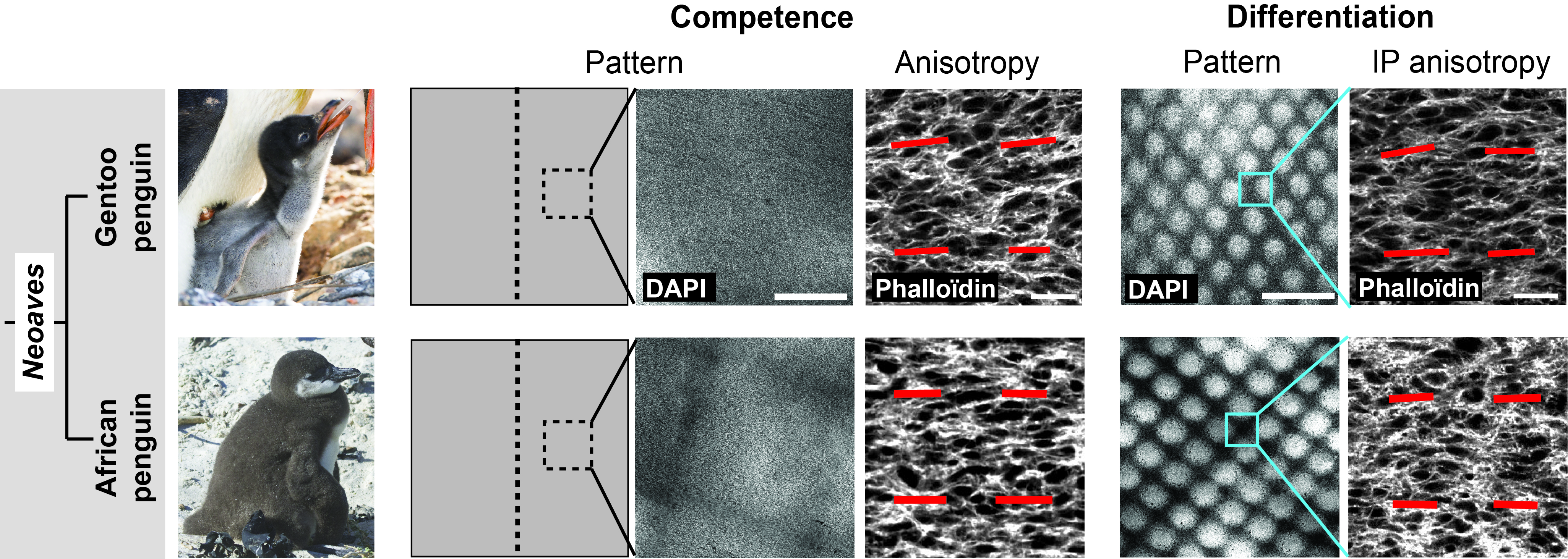

Supplement: S15 Fig — Left panel: African and Gentoo penguins are closely related Neoaves species. Middle panels: At competence stage in both species, 10× confocal views of DAPI stains and corresponding schematics showing the location of images in flat skins (see Fig 3; scale bars: 500 μm) and 100 μm2 magnifications of 40× confocal views of phalloïdin stains in inter-primordia regions (right scale bars: 20 μm), no pattern was visible. Right panels: At differentiation stage, African penguin skins displayed pattern geometry and dermal cell anisotropy (views correspond to blue squares) identical to those of the Gentoo penguin (and see Figs 2 and 3). Photo credits: Raphaël Sané (www.raphaelsane.com, Gentoo penguin) and Alain Bidart (www.alainbidart.fr, African penguin). (JPG) [file pbio.3001807.s015.jpg]

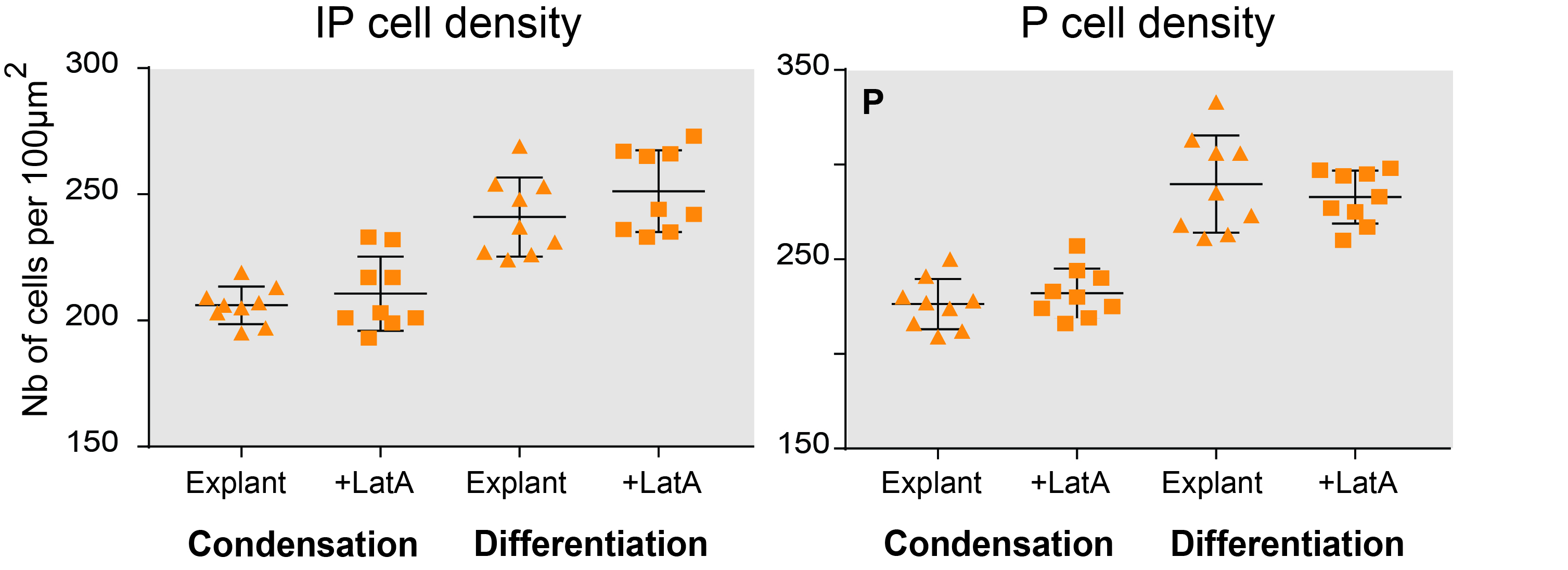

Supplement: S16 Fig — Quantifications of dermal cell density normalized to 100 μm2 areas at 3 different positions along the first formed row at condensation and differentiation stages showed no significant change between control (n = 3) and drug-treated (n = 3) Japanese quail explants in the inter-primordia region (left graph; IP; unpaired 2-tailed t test, p = 0.4076 at condensation stage and 0.1927 at differentiation stage) and primordia region (right graph; P; p = 0.3758 at condensation stage and 0.4895 at differentiation stage). The data underlying this figure can be found at 10.5281/zenodo.7006365. (JPG) [file pbio.3001807.s016.jpg]

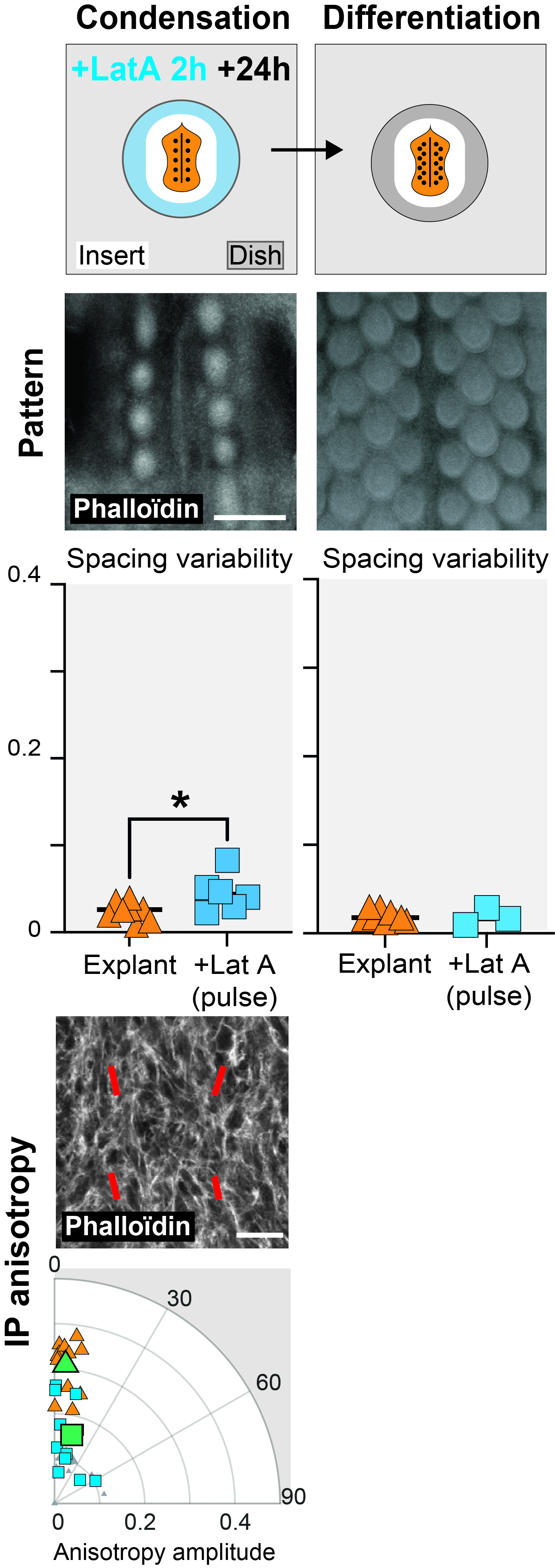

Supplement: S17 Fig — Japanese quail skin explants prepared at competence stage (in orange) were treated with low doses of Latrunculin A (LatA, in blue) during 2 h and cultured to condensation stage (+24 h; left panels) or differentiation stage (right panels). Quantifications of primordia spacing variability on phalloïdin-stained control flat skins (explant; triangles) and LatA-treated cultured explants (+LatA; squares) showed that the pulse of LatA only transiently modified pattern fidelity: it was lower at condensation stage (n = 8 explants; n = 6 +LatA; unpaired 2-tailed t test; p = 0.0307) but had entirely recovered at differentiation stage (n = 9 explants; n = 3 +LatA; unpaired 2-tailed t test; p = 0.7775). Confocal views and respective quantifications (as described in Fig 3) show that at condensation stage, pulsed drug treatment efficiently reduces cell shape anisotropy compared to control explants. Small data shapes are individual values, large data shapes (in green) are averaged values. The data underlying this figure can be found at 10.5281/zenodo.7006365. Error bars: mean with standard deviation. Scale bars: 500 μm (primordia pattern), 20 μm (anisotropy). (JPG) [file pbio.3001807.s017.jpg]

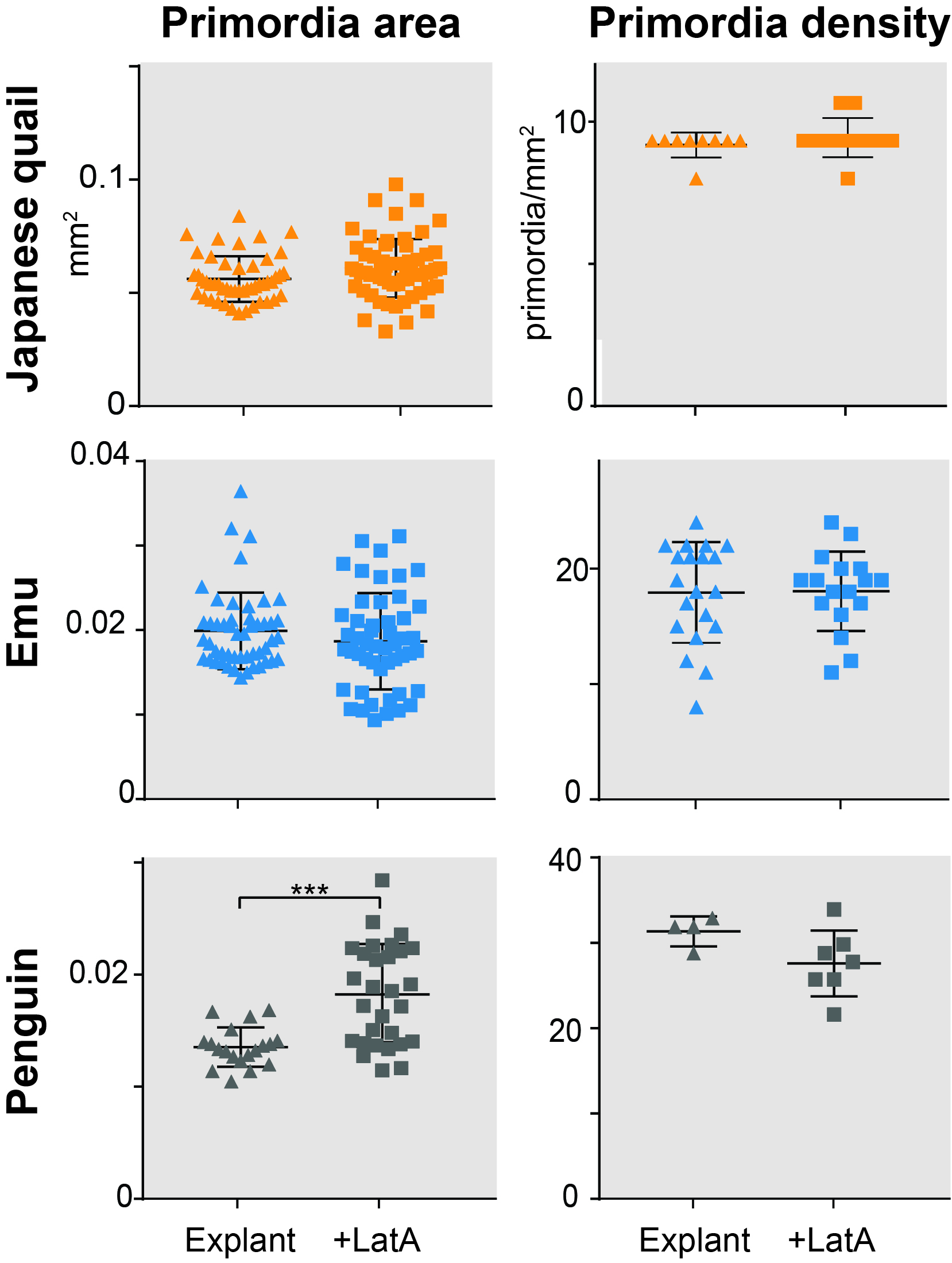

Supplement: S18 Fig — Left graphs: Primordia size (in mm2) did not change between control and drug-treated explants at differentiation stage of emus and Japanese quail (unpaired 2-tailed t tests; p = 0.2363 and 0.1910) but was significantly increased in drug-treated African penguin explants (p < 0.0001). Right graphs: Primordia density (in primordia/mm2) did not change between control and drug-treated explants in the 3 species (p = 0.9341, 0.3369 and p = 0.1054). The data underlying this figure can be found at 10.5281/zenodo.7006365. Error bars: mean with standard deviation; significance of statistical tests is shown with stars. (JPG) [file pbio.3001807.s018.jpg]

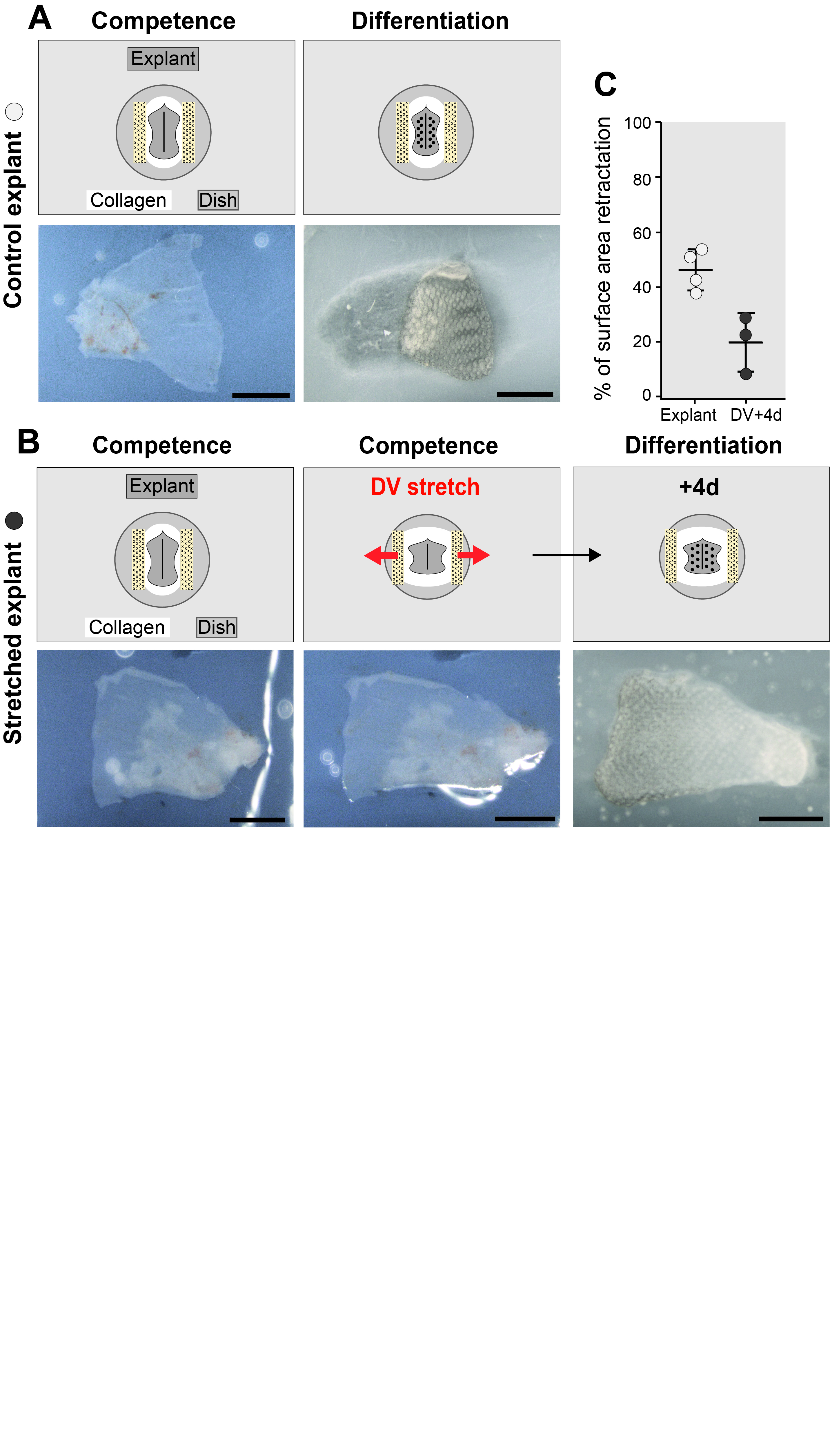

Supplement: S19 Fig — (A) Penguin explants were placed on collagen gels (in white) on Petri dishes (in gray) at competence stage (left panels) and cultured to reach differentiation stage (i.e., after 4 days; right panels). (B, C) In penguin explants prepared at competence stage (left panels) on which we applied controlled directional stretch (middle panels and see Fig 6), we observed a recovery of culture-induced retractation (right panels in B and quantifications in C; explants, white dots, n = 4; stretched explants, black dots, n = 3). The data underlying this figure can be found at 10.5281/zenodo.7006365. Scale bars: 2 mm. (JPG) [file pbio.3001807.s019.jpg]

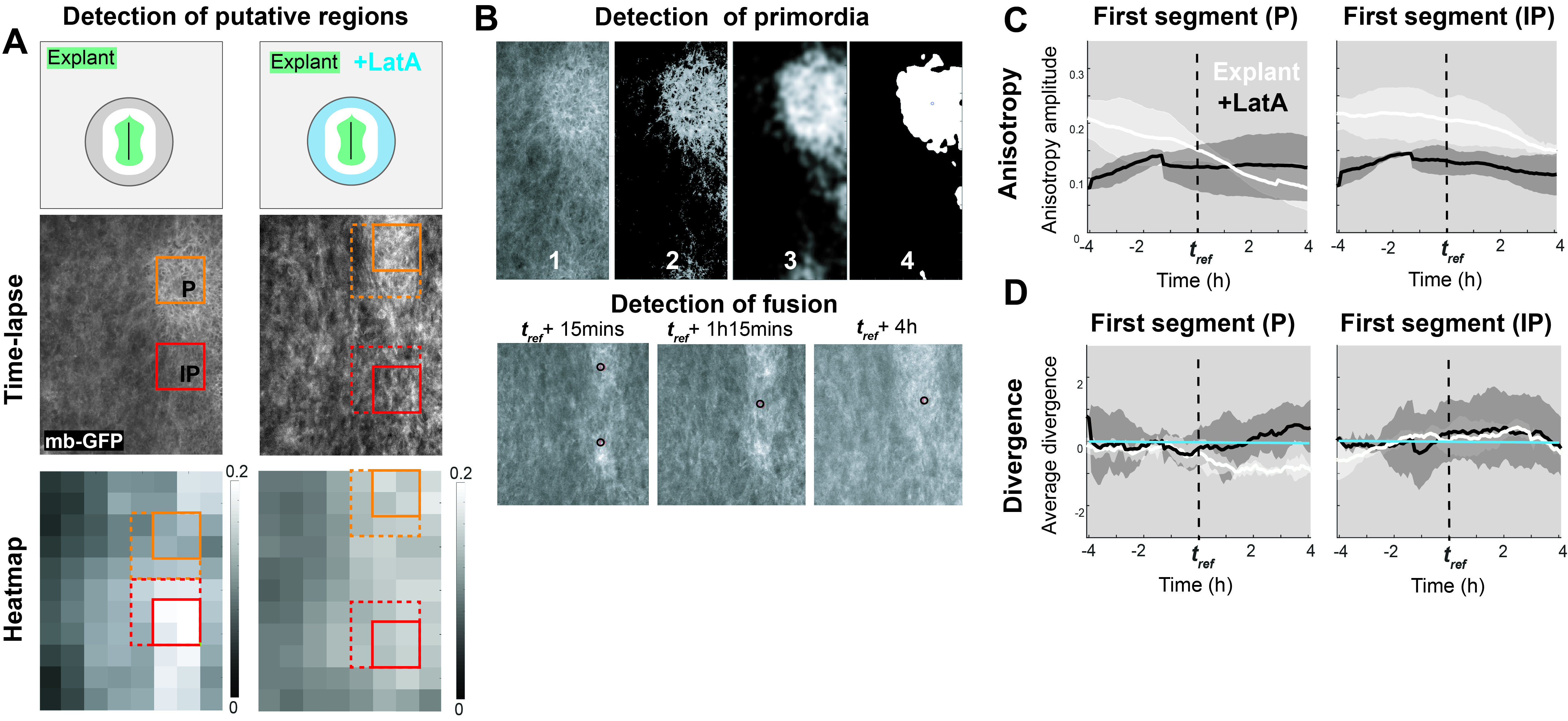

Supplement: S20 Fig — (A) Skin explants of membrane GFP (mb-GFP) Japanese quails were cultured as described in Fig 7. Snapshots of time-lapse confocal movies on control (n = 3) or Latrunculin A-treated (+LatA, in blue; n = 3) mb-GFP skin explants were computed to produce heat maps of average cell anisotropy amplitude per interrogation box (shades-of-gray squares). Over the course of each movie, putative inter-primordia (IP; red squares) and primordia (P; orange squares) regions were defined as the 4 interrogation boxes (for evolving anisotropy amplitude; full lines) or 9 interrogation boxes (for PIV analyses; dotted lines) with respectively maximal and minimal average anisotropy (see Materials and methods). (B) On time-lapse movie images (1) an algorithm detecting brightest pixels, (2) then applying Gaussian smoothing (3) was used to automatically identify putative primordia (4) and track their positions along the antero-posterior axis through time (see Fig 7I–7K). In bottom panels, snapshots of a time-lapse movie show that the algorithm first automatically detected 2 primordia (black circles) at tref+15min, then only one at tref+1h15min and tref+4h, thereby evidencing a fusion event. (C) Quantifications of dermal cell anisotropy amplitude within automatically defined IP and P regions of first-formed segments showed it significantly decreased through time (in hours to tref, marked with a black dotted line) after drug treatment (in black) compared to control conditions (in white; unpaired 2-tailed t test; p < 0.0001 for both IP and P). (D) Quantifications of the divergence of the vector field of dermal cell movement averaged within IP and P (the blue line represents value 0 at which there is no contraction or extension) showed that contraction in the primordium region, occurring 2 h after tref, decreases upon LatA treatment (in black) compared to control explants (in white). The data underlying this figure can be found at 10.5281/zenodo.7006365. (JPG) [file pbio.3001807.s020.jpg]

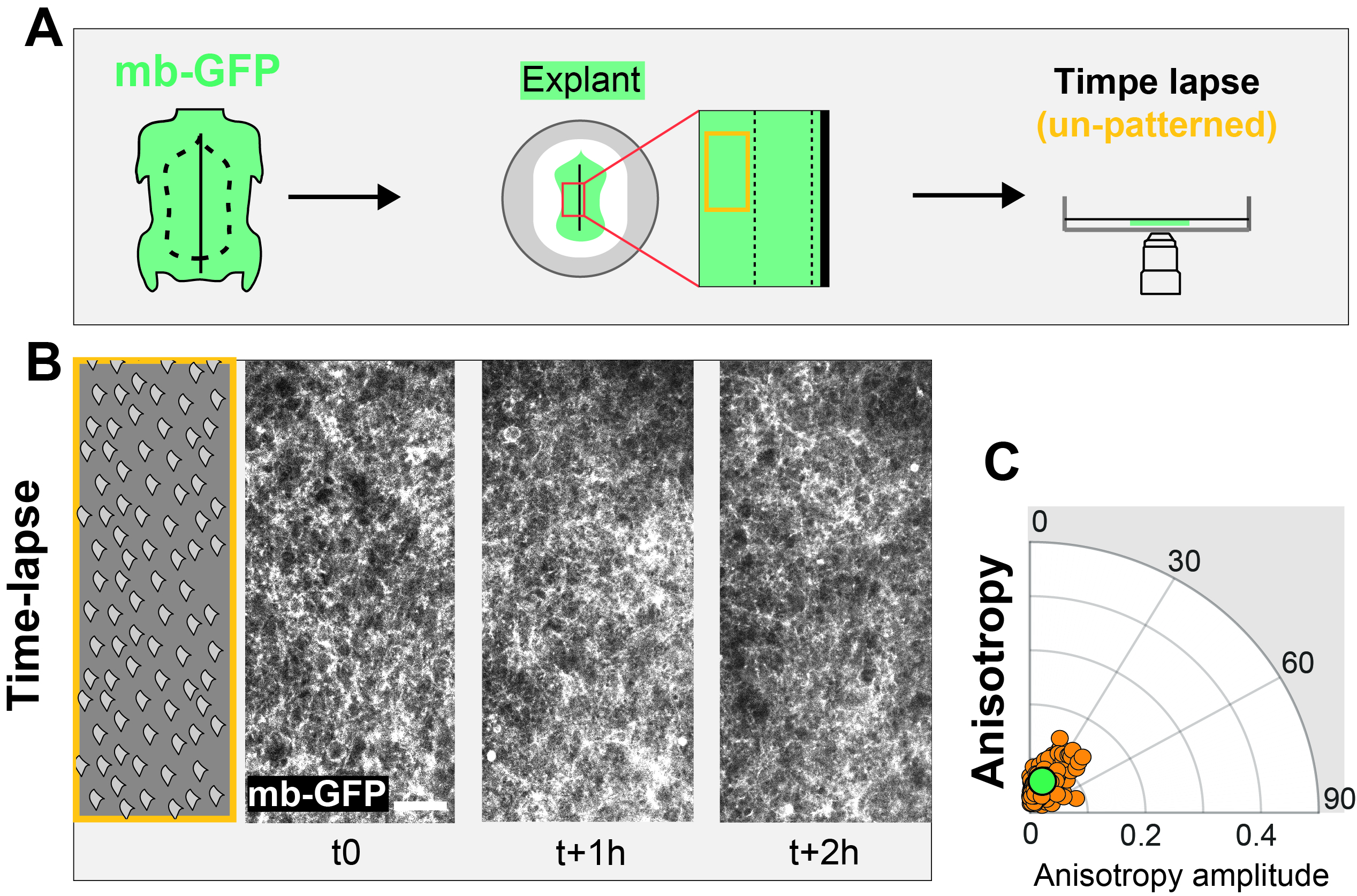

Supplement: S21 Fig — (A) To perform time-lapse experiments in un-patterned regions (in yellow), dorsal skin explants of membrane-GFP (mb-GFP) Japanese quails were placed dermal side down on nitrocellulose filters for confocal imaging (see Materials and methods). (B) Snapshots at t0 (and corresponding schematic), t+1 and t+2h of a time-lapse confocal movie in the un-patterned region. (C) Quantifications of cell shape anisotropy as described in Fig 3 show that dermal cells in the un-patterned region are isotropic. The data underlying this figure can be found at 10.5281/zenodo.7006365. Scale bar: 50 μm. Small dots are individual values; large dots (in green) are averaged values. (JPG) [file pbio.3001807.s021.jpg]
