# Supplementary material for: Cell shape anisotropy contributes to self-organized feather pattern fidelity in birds
Source: PLoS Biol. 2022 Oct 10;20(10):e3001807. doi: 10.1371/journal.pbio.3001807 (PMC9584522; doi:10.1371/journal.pbio.3001807)
Supplement: Attachment — Submitted filename: Response to reviewers_Curantz et al_Revised.pdf [file pbio.3001807.s025.pdf]

RESPONSE TO REVIEWERS

We thank the reviewers for thoughtful comments on the manuscript. We provide below a point-by-point answer detailing all modifications (in blue). In sum, we added (1) novel imaging experiments showing that cell motility increases upon morphogenesis, strengthening our conclusions that it represents an important initial condition of the self-organizing skin system, (2) pulse-experiments showing that the drug-mediated effect on cell shape anisotropy is linked to a temporal control of pattern stabilization, and (3) quantifications of explant retractation in control and stretched conditions. In addition, we extensively modified the text and figures to air the manuscript, clarify statements (in particular those related to the implication of cell motility), and extended the discussion.

Rev.1:
Spatial arrangements of cells into patterns often arise when stochastic changes in cell properties are stabilized and amplified. It is therefore unclear how reproducible self-organization, which is crucial to organogenesis and organism survival, is achieved across individuals and species. A key property in tissues undergoing patterning is tissue anisotropy. This study uses a cross-species comparative approach to address the role of dermal anisotropy during avian feather follicle patterning. The authors first show that birds of distinct clades that display variable regularity of their feather primordia arrays also have corresponding differences in anisotropy--higher anisotropy generally correlates, in time and between species, with the appearance of more regular patterns. The authors perturbed anisotropy pharmacologically and mechanically, and saw that a shift toward isotropic organization using actin polymerization inhibitor LatA and uniaxial stretch orthogonal to existing tissue orientation caused more disordered follicle patterns. Conversely, forced anisotropic organization using stretch restored pattern order in a disordered follicle pattern/isotropic dermis condition. Finally, live imaging showed that LatA caused higher cell motility and destabilized the shapes and dynamics of primordia condensates. Altogether the authors propose a model where tissue anisotropy constrains cell motility and limits the positioning of primordia into ordered patterns, while isotropic arrangement allows for free movement into randomized arrays.

This study adds to a growing body of knowledge concerning physical mechanisms of spatial pattern morphogenesis. The authors define an intriguing and somewhat unexpected relationship between anisotropy and motility on the scale of individual cells to explain a tissue-level phenomenon of pattern fidelity. This is an important contribution, as links between tissue and cell-scale physical processes often remain poorly defined as we learn more about the role of mechanics in development. Furthermore, because the authors take advantage of species-specific differences, findings may be considered in an evolutionary context, and could inform potential functional consequences of feather patterning for avian survival and adaptation. On a technical level, the authors use a variety of cross-disciplinary techniques and robust quantifications with many replicates to support their findings. While this submission is certainly appropriate for PLoS Biology, being an interesting study, and potentially making a significant contribution to the field, several of the conclusions need to be better substantiated prior to acceptance:

Major Critiques:

1. The part of the model concerning cell motility comes only from an experiment using Latruncilin A, which inhibits actin polymerization and presumably directly affects migratory properties. Even though the LatA effect on cells is the opposite of what one would expect (lower motility), it is unclear whether isotropic organization and high motility are independent downstream effects of perturbing actin, rather than motility depending on cell shape. Experiments demonstrating that stretch-induced anisotropy reduces motility, and/or that cells in isotropic dermal tissues from paleognaths are more motile than in quails or chickens, are needed to lend credibility to this part of the model.

We agree with the reviewer's comment that causality between cell shape anisotropy and cell motility is not formally demonstrated. Our goal is rather to show that both cell behaviors constitute initial cellular properties that shape the self-organization of the competent skin.

Unfortunately, suggested experiments are not feasible within the time allowed for reviews:
- Demonstrating that explant stretching reduces cell motility requires combining live imaging with stretching experiments. While we aim at developing live-imaging protocols compatible with mechanical perturbation experiments, it is not currently possible to perform stretching on insert membranes used for live imaging, and conversely, to perform live-imaging on collagen gels used to apply stretch.
- Recording motility in *Paleognathae* is also not currently feasible due to the absence of transgenic strains of ostriches and emus (though we aim at developing non-fluorescent live imaging using second harmonic techniques). In addition, paleognaths are seasonal breeders and fertilized eggs are available only in April/May for ostriches and November/December for emus.

To address the reviewer comment, we performed two additional experiments.

- First, we performed additional live-imaging experiments to record cell motility in the naïve, un-patterned skin regions of mGFP Japanese quails, in which cells are isotropic. We had previously recorded cell motility only in the first patterning segment, in which cells become anisotropic. We found that cells of the non-competent dermal mesenchyme move randomly even in absence of morphogenesis, but more slowly than in the first competent segment. Together with our previous data, these novel experiments show that primordia patterning is preceded by an increase in cell motility that is both concomitant to, and limited by, the increase of cell shape anisotropy.
These results are now displayed in Fig 7 (formerly Fig 6) and a novel Fig S21. The schematics describing the live-imaging procedure, the abstract (line 32), the text (lines 273-278, 284-285, 294-299), and figure legends have been modified accordingly.

- Second, we further explored the effect of a Latrunculin A drug treatment on patterning dynamics and pattern fidelity. To do so, we applied Latrunculin A at competent stage in Japanese quail explants in a 2-hour pulse instead of continuously throughout pattern formation. This impaired early dermal cell anisotropy, but pattern fidelity was only transiently affected: it was lower than in un-treated explants at condensation stage, but had recovered at differentiation stage. These results strengthen our conclusion

that cell shape anisotropy is involved in timely dynamics of primordia emergence. We describe these new results in the text (lines 223-231) and in a novel Fig S17.

In light of the reviewer comments and these complementary results, we modified the result section to remove statements that (1) Latrunculin A directly affects cell motility, and that (2) cell shape anisotropy controls cell motility, instead extending the discussion section in which we suggest so. Specifically, we hypothesize that anisotropy-driven changes in the actomyosin network result in modifications of tissue material properties, themselves influencing cell motility (discussion section, lines 392-405).

2. The proposed causal relationship between constrained vs unconstrained movement and an ordered vs. disordered primordial array (respectively) is not well demonstrated in this study alone. The addition of simple 2D mathematical model or, at the very least, references to established works that support this effect of motility on pattern order is needed. Additional experiments decoupling anisotropy from motility in the system would also strengthen the argument, though these would be understandably difficult to do.

We previously produced a mathematical model combining reaction-diffusion, chemotaxis and logistic cell proliferation that successfully reproduced natural variation in dynamics of primordia pattern emergence (Bailleul et al., 2019; #26). To address the reviewer comment, we varied model parameters that indirectly influence cell motility. We found that “cellular diffusion D_n” or “chemotaxis sensitivity khi” influence the sharpness of the individualization of dotted-shapes representing primordia (see Figure below). These numerical predictions are consistent with our experimental data, however we felt they did not significantly strengthen our study as the model does not take into account cell shape anisotropy, and parameter variation is therefore difficult to interpret biologically.

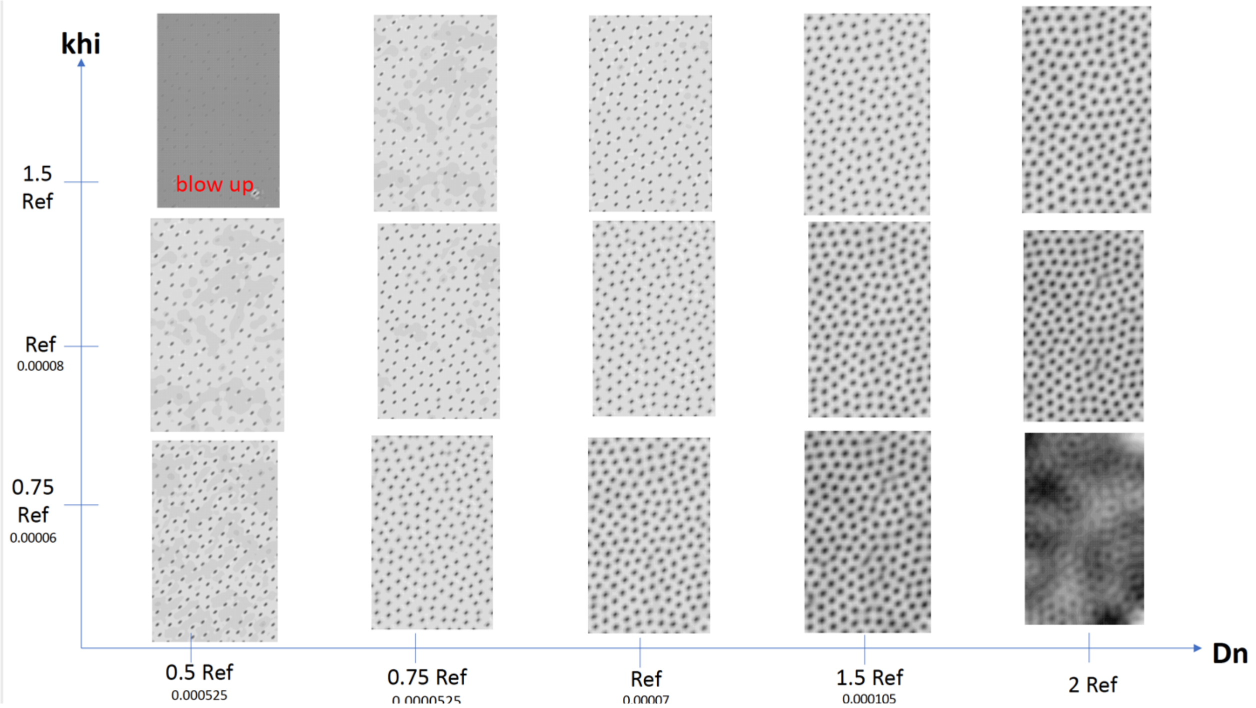

To the best of our knowledge, no studies have thus far formally demonstrated a link between mesenchymal cell motility and pattern fidelity. As mentioned above we extensively reworked the discussion of results related to cell motility (lines 385-405).

In addition, we clarified the implication of tissue-scale movement (i.e. primordia displacement) to avoid confusion with cell-scale movement (results section, paragraph related to Fig. 7). More generally, we rephrased the manuscript to better emphasize our focus on cell shape anisotropy as a causal modulator of tissue-scale pattern order.

3. There are a few results that seemingly contradict the notion that anisotropy leads to less spacing variability in the primordia array (and vice versa), which is central to the paper's findings. 1) Comparing Fig. 3B and C (emu vs quail), the distributions and means of amplitudes are very similar, but the spacing variabilities appear different between species—as different as penguin flat vs explant in Fig. 3D. 2)

This apparent contradiction is due to temporal aspects of pattern fidelity acquisition: when present, cell shape anisotropy is transient, and its effects on pattern outcomes are observed at a stage when it is not visible any more. Specifically, at competence and condensation stages, cells are isotropic in the emu and anisotropic in the quail, while later at differentiation stage (when we quantified the resulting pattern fidelity, as displayed in previous Fig. 3B/C), cells are isotropic in both species (i.e. anisotropy drops in the quail).
To clarify, we added images and quantifications of cell shape anisotropy in the Japanese quail at competence and condensation stages (Fig 4E, formerly Fig 3B). We modified the schematics (Fig 4A). We extensively modified the text in the corresponding paragraph to emphasize the link between temporal dynamics of cell shape anisotropy and resulting pattern fidelity (lines 175-184).
To air the modified figure, we moved all tests performed in emus to a novel supplementary figure (Fig S14).
We modified Fig 4 and Fig S14 legends accordingly.

In Fig. 3D, the penguin explant primordia pattern shows higher spacing variability, but the cell arrangement still appears anisotropic, just oriented orthogonally to the penguin flat skin in Fig. 2 (penguin explant appears nearly as AP oriented as the Fig. 2 Cd stage Japanese quail).

In penguin explants, dorso-ventral cell shape anisotropy is lost globally, however we observed variability in its amplitude and orientation depending on location: at places, cells are isotropic or anisotropic along the antero-posterior axis, while in other regions, dorso-ventral anisotropy remains visible (though with lower amplitude values than *in vivo*). This spatial variability is reflected in the distribution of measurements in polar plots.
We added this information in the result section (lines 193-204), and modified the figure to show high magnifications of the dermis on two locations of the penguin explant representative of local cell shape anisotropy differences (now Fig 4K, L).
For consistency, asterisks marking the corresponding location of phalloidin-stained confocal views were added to images of skin explants in Fig 4-6 (formerly Fig 3-5).

2) In Fig. 5C, though apparently not significant, the DV+48hr amplitudes appear more DV oriented than the control, but have significantly more spacing variability.
Though the authors somewhat acknowledge these findings, they should include a more complete discussion/justification in the text of how these apparent contradictions affect the final model.

As described in the response to comment #3-1 above, this contradiction stems from the fact that results related to Fig 6F (formerly Fig 5C) are shown at differentiation stage, when cell shape anisotropy has normally dropped. In addition, cell shape anisotropy in stretched explants at both competence and differentiation stages becomes slightly oriented along the dorso-ventral axis (i.e. orthogonally to *in vivo* and unperturbed conditions).
As above, we modified the text related to Fig 6 to clarify the link between temporal dynamics of cell shape anisotropy and resulting pattern fidelity (lines 252-259).

Minor Critiques:

1. Some stained fluorescent images are either too small or too dim, eg. Fig. 1B, Fig. 2A, Fig. S5

We split the previous Fig.1 in two novel figures. Novel Fig.1 shows the emergence of one primordium in the developing epidermis and dermis. Novel Fig. 2 shows species-specific dynamics of primordia array emergence. We modified figure legends accordingly.

We increased brightness in images of Fig 3A (formerly Fig 2A) and Fig S5.

2. Fig. 3-5, S12 individual data points in anisotropy amplitude plots are way too small (can't distinguish shapes). This may be a personal preference but the shapes are inconsistent, eg. explant is triangle in Fig. 3 but control is triangle in Fig. 4.

We increased the size of data point shapes in all figures.

The term “control” was confusing as it englobed two different conditions (i.e. *in vivo* observations or un-perturbed *ex vivo* explants). We kept the consistent use of rounded shapes for *in vivo* conditions (flat skins), triangles for un-perturbed *ex vivo* conditions (explants) and squares for perturbed *ex vivo* conditions (explants + drug treatment or stretch), but changed the term “control” to either “flat skin” or “explant” in Fig 4-6 (formerly 3-5) and novel Fig S13, S14 (formerly S10,12).

3. Fig. 6, S14 black and white curves and data points need a legend somewhere specifying explant/explant + LatA. Fig 5D box plots need x-axis labels.

We added color-coded explant/+LatA legends in graphs of Fig 7 (formerly Fig 6) and Fig S20 (formerly S14).
We added the missing x-axis labels in Fig 6J, K (formerly Fig 5D).

4. Fig. 6B,C plots of Primordia position vs. Time are somewhat hard to interpret. Perhaps something like distance between primordia or primordia aspect ratio over time would be more clear ?

Plotting the distance between primordia would not take into account fusion events occurring in drug-treated explants, and plotting primordia aspect ratio would be subject to ascertainment bias (i.e. in deciding whether a group of cells represents a nascent primordia), which we avoided using unbiased custom software automatic detection.

To ease the interpretation of plots in Fig 7F (formerly Fig 6), we added a schematic next to graphs that helps visualizing how data points correspond to an antero-posterior tracking of nascent primordia displacement. We modified Fig 7 legend accordingly.

Rev. 2:

Feather primordia form exquisite patterns. In emu, chicken, finch, penguin, etc. there are differences in the bud size, spacing, the way they form (propagation versus simultaneously), yet in each case the basic periodic feather patterns form. Therefore, the authors ask an interesting question on how the pattern fidelity in the dorsal skin of birds are maintained and hoping to use this approach to decipher the fundamental principles of feather pattern formation. In this manuscript, then then focus on the roles of cell shape anisotropy and hypothesize that it is important for fidelity of feather pattern formation. They did some substantial analyses based on this hypothesis. This comparative pattern formation study is interesting: they compare cell shapes and the emergence of feather patterns. Some whole mount molecular expression figures are beautiful, but some are unclear.

As described above, we reworked figures as follows:
- we split Fig. 1 in two. In the novel Fig 2, the relative size of pictures showing primordia emergence and species-specific dynamics of primordia pattern formation is thereby increased.
- we split Fig. S4 in two, thereby increasing the relative size of pictures showing epidermal (novel Fig. S4) and dermal (novel Fig. S5) densities.
- we increased data point shape sizes, and modified/added explanatory schematics in Fig 4-7 (formerly Fig 3-6).

The writing is dense and should be improved to make it clearer for general readers to follow.

To air the manuscript, we extensively modified/extended the introduction, the results section (in particular in paragraphs describing experimental tests of cell shape anisotropy corresponding to Fig. 4-7), and the discussion (see details below).

Overall, the conclusion is cell shape anisotropy can constrain avian dorsal feather pattern fidelity. Yet, many parameters can affect cell shape anisotropy, and these are not explored.

We dedicated a paragraph of the discussion to this point (lines 423-435): the embryonic origin of cell shape anisotropy may be linked to the size and shape of the patterning space, to contractile cell properties, to externally or intrinsically generated anisotropic tensile forces, etc. We cited corresponding references (#48-50), and a sentence stating how further comparative and functional work exploring will be necessary to explore these possibilities.

The citation and discussion of the literature need improvement. Many papers directly relevant to this manuscript are not cited properly and discussed - in fact some findings in these literatures are in line with the concept here. So, it is good for the whole field if authors can integrate them and make a well-synthesized discussion. There are also gaps which should be addressed.

We extended the discussion and added relevant references mentioned by the reviewer as described point-by-point below.

In addition, we integrated a recent publication by the Shyer laboratory (Palmquist et al. 2022; #13) demonstrating that dissociated mesenchymal cells from the avian dermis autonomously acquire cell shape anisotropy and produce primordia-like patterns. This work is complementary to ours as it shows that contractile forces drive large-scale self-organized pattern formation.
Our conclusions however differ with regards to the role of cell shape anisotropy, viewed in this study as required for pattern formation. By comparing several species, we demonstrated that cell shape anisotropy is *not* required for self-organization (patterns emerge in isotropic mesenchymes such as those of emus/ostriches or of drug treated skin explants). Instead, cell shape anisotropy potentiates the developing skin tissue such that it produces high fidelity patterns.

In light of these findings, we now discuss how our work provides information on the robustness of self-organized primordia patterning (lines 334-353) and the potential link between cell shape anisotropy and cell contractility in the avian dermis (lines 411-414; 423-425).

Major comments:

1. About periodic formation of feather germs, pattern fidelity:
In an earlier paper, it is shown that when dissociated chicken dermal cells are recombined with epithelium, the dissociate dermal cells mediate simultaneous formation of feather primordia. These newly formed buds are always of the same size. But the bud number varies, and is the function of the number of dermal cells added. With the increase bud density, the inter-bud spacing decreases. Although "pattern fidelity", "cell shape anisotropy" is novel in this manuscript, this classical paper is a beautiful example of converting a pattern with fidelity in the chicken in vivo to a non-fidelity pattern in vitro due to the disruption of dermal cell anisotropy (and something else). The work is in line with the authors' model and should be cited and discussed together.

Jiang, T. X., Jung, H. S., Widelitz, R. B., Chuong, C. M. (1999). Self-organization of periodic patterns by dissociated feather mesenchymal cells and the regulation of size, number and spacing of primordia. Development, 126(22)4997-5009.

We now mention these results in a novel paragraph of the discussion focusing on the role of cell shape anisotropy in the timely acquisition of competence and pattern fidelity (lines 354-376). We added the corresponding reference (#41).

2. Cell shape anisotropy. This term refers to cell properties at the morphological level.

Proliferation control, cell migration, etc. It is ok to focus the question on this level, where one might expect a global level cell shape anisotropy and local level cell shape anisotropy. Many biophysical and biochemical factors can affect cell shape anisotropy. Please discuss the inputs of these underlying factors regulating cell shape more clearly. In addition to Eda, FGF 20 (Headon, PLOS bio) which was cited, Lin et al., 2009 also discussed this issue and should be cited.

Lin, C. M., Jiang, T. X., Baker, R. E., Maini, P. K., Widelitz, R. B., Chuong, C. M. (2009). Spots and stripes: Pleomorphic patterning of stem cells via p-erk-dependent cell chemotaxis shown by feather morphogenesis and mathematical simulation. Dev Biol, 334(2)369-382.

We now cite the abovementioned study (ref #29) in a novel part of the discussion speculating on the relationship between cell shape anisotropy and cell motility (lines 390-392), and more generally, mention previously-identified biochemical candidate patterning factors in the introductory paragraph to Fig 1 (lines 80-82) and in the discussion (we suggest a likely interaction of cell shape anisotropy with molecular network responsible for primordia self-organization; lines 413-417).

3. About bud forming simultaneously or progressively:
Chuong group has explained their view in Inaba et al., 2019. It stated Turing patterning can occur locally, if threshold is achieved (as seen in Emu). Or, global events can add to lower the threshold that triggers Turing patterning. Cell shape anisotropy could be part of the unknown global events mentioned in the Inaba et al paper. While these authors may have other views. Whatever their views are, the concept of this relevant paper should be discussed and reconciled in the discussion.

Inaba M, Harn HI, Chuong CM. Turing patterning with and without a global wave. PLoS Biol. 2019 17:e3000195.

As for comment #1, we now extensively discuss this study in a novel paragraph of the discussion focusing on the role of cell shape anisotropy in the timely acquisition of competence and pattern fidelity (lines 354-376). We added the corresponding reference (#40).

4. In Figure 3, in ex vivo cultures, primordia density increased and primordia size decreased, consistent with the marked reduction in explant size in this species. The data for spacing variability was shown. Please provide further quantification of the decreased explant size.

We quantified explant retractation in Japanese quail, emu and penguin explants at competence stage (i.e. upon explant preparation) and at differentiation stage (when pattern fidelity is recorded) by manually outlining the surface of explants using the Fiji software polygon selection tool, and recording the obtained area value. We showed that while Japanese quail and emu explants only slightly decrease in size through time, penguin explants shrink by ~50% of their surface area. These results are now shown in novel Fig S12 and detailed in the main text (lines 176-179, 185, 193-194).

In addition, we quantified retractation in penguin explants after stretching and showed they recovered from culture-induced shrinking. These results are shown in novel Fig S19 and detailed in the main text (lines 262-264).

Quantification procedures have been added to the methods section.

Also, please explain more clearly why this led to increased feather numbers with smaller size.

We quantified primordia density by counting the number of primordia in 1mm^2 at differentiation stage (see methods). Penguin explants strongly retract over time, thus as quantified, the final density of primordia appears greater. In addition, on these condensed explants, primordia almost touch each other. Lack of developing space may thus explain their smaller sizes.
We added an explanatory sentence in the result section (lines 195-197).

Minor comments
1. Title: probably better to specify feather to be clearer what this paper is about. "Feather pattern fidelity" instead of "pattern fidelity".

We modified the title.

2. Fig. 1B and Fig. S1

- The orientation of Fig. 1B and Fig. S1 should be clearly marked.

To clarify, we added a 10X confocal image in Fig 1A illustrating overall primordia pattern geometry, and showed its orientation by linking it to the schematics (red lines/square). We also added to this image a red square (a) clarifying the orientation of higher magnification 40X views shown in Fig 1B.
We mentioned the corresponding location and orientation of all images of Fig S1 in its legend.
We modified figure legends accordingly.

- How the placement of the broken white line drawn was decided is not clear. There does not appear to be a clear border and the line can be arbitrary. Perhaps they can draw a half a circle on one figure and leave the other to show there is a boundary.

In Fig 1A and Fig S1, we kept white circles in images of the epidermis and removed them from images of the dermis, in which nascent primordia are clearly visible. Instead, we pointed them on phalloidin-stained pictures with white arrowheads. We modified the figure legend accordingly.

- White arrowheads point to beta-catenin. It is asymmetrical. Is this asymmetry position consistent in all buds?

This asymmetry is consistent in all primordia at differentiation stage in the Japanese quail. It is possible that it is linked to the future antero-posterior orientation of growing feather follicles. While this is an interesting observation, it is not directly linked to our conclusions, and for clarity, we decided not to mention this detail in the main text.

3. Fig.1D, the two right columns, the position of the midline should be indicated and the number of rows away from the midline should be stated. Beta-catenin and bud sizes are the same in many birds, but different in emu. Why?

We added dotted lines representing the position of the dorsal midline (dm) in the novel Fig 2 (formerly Fig 1C, D). We added boxes showing the position of the first-formed primordia row (fr) at condensation stage (all pictures were taken when only the first row was visible) and differentiation stage. We modified the figure legend accordingly.

The size of primordia is indeed strikingly smaller in emus compared to other species. This may be linked to its lack of flight abilities (i.e. during evolution, selection may have been relaxed on the maintenance of an optimal primordia size necessary for the production of functional feathers). While this is an interesting observation, related evolutionary speculations are out of the scope of this study and we chose not to discuss this point in the present manuscript.

In penguin, in Co stage, beta-catenin appears to be a circle, and in Cd stage, beta-catenin appears to be on one side only?

ß-catenin transcripts indeed appear asymmetrical at condensation stage. This may be linked to mechanisms of feather follicle formation unique to in penguins, consistent with the drastic modification in the structure of their feathers. However, the ß-catenin protein is homogeneously distributed (see Fig S1). Studying primordia differentiation in penguins would be exciting, however out of the scope of this study.

4. Fig. 2. and Fig. S5, since the study is about the shape, the position of the midline, and the position of the panel within the body (how many rows lateral to the midline) should be marked clearly in a simple schematic drawing. Within the panel, the location of the bud should also be pointed out. Or do the red bars mark the boundary of the primordia?

For each phalloidin-stained 40X confocal picture in Fig 3, Fig S6-8, and Fig S9 (formerly Fig 2 and Fig S5, 6), we added a corresponding schematic showing the position of image within or between primordia (dotted squares) and the dorsal midline (dm; dotted line).
In the cases of the Japanese quail, domestic chicken and penguin, we also added boxes showing the position of the first segment (fs) at competent stage, and a “first-row” (fr) legend at condensation stage. We modified figure legends accordingly.

If the midline is the same for all, the dermal cells appear to be anterior-posterior oriented in the chicken, but medial-lateral oriented toward the midline in the penguin. But the skin from both birds form buds. Is this true?

Cell shape anisotropy is indeed oriented along the antero-posterior axis in Japanese quails, domestic chicken and zebra finches, but strikingly, along the dorso-ventral axis in the penguin (see results section, lines 150-160).

5. Fig. S1. Are all buds from the same position of the embryos? Why Zebra Finch shows a different configuration in Cd specimens? I understand it is not easy to get all

specimens to be in the same positions. But this has to be made clear so the findings can be useful to others.

All primordia are in the same position, which we now indicated in the Fig S1 legend. The seemingly angled position of primordia in zebra finch embryos is due to higher wrinkling of flat skin specimens in this species.

6. Fig. S4, It is valuable to have the cell density data. Again, the orientation of the panel in the context of the whole embryo has to be accompanied by a schematic drawing.

In novel Fig S4 and S5 (formerly Fig S4), we added schematics for each cell density picture at competence stage indicating the position of the dorsal midline (dm). In the case of Japanese quail, domestic chicken and zebra finch embryos, we added boxes showing the location of the first competent segment. We modified figure legends accordingly.

7. Fig. S8. The two columns under Co. Are they of the same orientation? Is this what you are trying to show with the red bars?

Red bars in novel Fig S15 (formerly Fig S8) indicate the amplitude and orientation of cell shape anisotropy (see methods and Fig 3 legend). To clarify, we added for both bird species (1) schematics indicating the position of the image in the competent area of the skin, and (2) the location of 40X confocal views of phalloidin-stained skins at differentiation stage (blue squares).

8. In introduction and discussion, the following papers should be properly cited.

About the role of BMP, FGF in Turing periodic patterning of feathers, the first paper on this topic should be cited.

Jung, H. S., Francis-West, P. H., Widelitz, R. B., Jiang, T. X., Ting-Berreth, S., Tickle, C., Wolpert, L., Chuong, C. M. (1998). Local inhibitory action of Bmps and their relationships with activators in feather formation: Implications for periodic patterning. Dev Biol, 196(1)11-23.

We added the reference (line 82).

About whole mount beta-catenin in situ pattern and the shift of bud position during patterning:

Chuong, C. M., Yeh, C. Y., Jiang, T. X., Widelitz, R. B. (2013). Module based complexity formation: Periodic patterning in feathers and hairs. Wiley interdisciplinary reviews Developmental biology, 2(1)97-112.

We added the reference (lines 74, 82).
