# Supplementary material for: Cell shape anisotropy contributes to self-organized feather pattern fidelity in birds
Source: PLoS Biol. 2022 Oct 10;20(10):e3001807. doi: 10.1371/journal.pbio.3001807 (PMC9584522; doi:10.1371/journal.pbio.3001807)
Supplement: Attachment — Submitted filename: Response to reviewers_2.docx [file pbio.3001807.s026.docx]

**Response to reviewers**

Reviewer #2:

The revision has improved and many previous concerns are addressed properly. Authors ask an interesting question in an unexplored topic and has accumulated interesting observation to demonstrate a correlation between cell shape anisotropy and feather pattern fidelity.

Major:

Yet, a major concern I share with R1 is that the causality of cell shape anisotropy and pattern fidelity is not strongly established. Authors did try to test the relationship. But the use of latrunculin and biostretch are not super specific and they state they are not able to evaluate the mechanism at cellular (e.g., cell motility as asked by R1) or molecular level in the allowed time frame. Both latrunculin and biostretch may cause changes other than cell shape anisotropy. Thus, while the work is still beautiful and valuable, I suggest authors tune down the claim and acknowledge the possibility of other mechanisms. For example, in the title, authors may consider replace "constrains" with "contributes to" . It is good for the community and authors to leave some spaces for other mechanisms.

We modified the title according to the suggestion

Also, in the text, please adjust some wording. For example, line 168, "influence" is too strong. Also, please similar type of wording in other places.

167	These observations suggested that

168 emerging properties of cell anisotropy influence the self-organization of primordia such that

169 they result in arrays with species-specific fidelity.

Line 168: we replaced “influence” by “contribute to”

Line 204: we removed the word “strongly” (strongly suggest > suggest)

Line 239-240: we replaced “demonstrated that” with “associated”

Line 256: we replaced “demonstrating” with “supporting”

Line 268-269: we modified the sentence to tune down the general conclusion: “a key initial condition > an initial condition””; demonstrated (…) that it ensures > indicated that it contributes to”

Line 351-352: we modified the sentence to acknowledge the possibility that drug treatment and stretching cause several and concomitant changes in cellular dynamics (i.e., “though drug treatment or stretching may cause several cellular changes, our approach allowed us to link early cell shape anisotropy to final pattern fidelity”.

Minor:

Fig. 1. Panel C and D, the positions of the primordia. I suppose they are lined up anterior-posteriorly. Where is the midline? Please mark them.

The position of developing primordia is indicated with a red square marked “a” in A. We modified the Figure legend to clarify that the red square refers to all panels B, C, and D. Showing the midline on the picture would require much lower magnification, but we added a mention of image orientation in the legend (lines 583-588).

Also, in epidermis, in condensation and differentiation stage, why strong beta catenin staining is in the posterior and anterior primordia respectively?

At condensation stage, ß-catenin staining appears slightly stronger posteriorly due to a fold in the flat skin preparation. At differentiation stage, ß-catenin staining appears stronger anteriorly because the protein locates in the nucleus in this region of the primordia, transiently remaining in cell membranes in the posterior region. The process of ß-catenin translocation is indeed spatially dynamic; the biological cause of this spatial difference is not known. To acknowledge it, we added a sentence in the figure legend (lines 589-590).

Fig. 2C, Cd, Di should be defined in figure legends here.

Reference to abbreviations was added in the figure legend (lines 604-605).

On the differentiation panel of emu and ostrich, why the primordia are different in size between beta catenin and DAPI staining?

DAPI-stained primordia (i.e., in which cells are marked) consistently appeared slightly larger than *ß-catenin*-stained primordia (i.e., in which transcripts are marked) in these species. This may be due to a larger distribution of condensed cells than *ß-catenin* transcripts, or differences in detection levels between immunostaining and *in situ* hybridization in these species.
